# Specific Interleukin-1 Inhibitors, Specific Interleukin-6 Inhibitors, and GM-CSF Blockades for COVID-19 (at the Edge of Sepsis): A Systematic Review

**DOI:** 10.3389/fphar.2021.804250

**Published:** 2022-01-21

**Authors:** Ying Wang, Kun Zhu, Rulin Dai, Rui Li, Miao Li, Xin Lv, Qian Yu

**Affiliations:** ^1^ Department of Pharmacy, China-Japan Union Hospital of Jilin University, Jilin University, Changchun, China; ^2^ Center of Reproductive Medicine and Center of Prenatal Diagnosis, The First Hospital, Jilin University, Changchun, China; ^3^ Department of Neurosurgery, China-Japan Union Hospital of Jilin University, Jilin University, Changchun, China

**Keywords:** specific interleukin-1 inhibitors, specific interleukin-6 inhibitors, GM-CSF blockades, coronavirus disease 2019 (COVID-19), SARS-CoV-2, sepsis

## Abstract

Sepsis is a syndrome with high mortality, which seriously threatens human health. During the pandemic of coronavirus disease 2019 (COVID-19), some severe and critically ill COVID-19 patients with multiple organ dysfunction developed characteristics typical of sepsis and met the diagnostic criteria for sepsis. Timely detection of cytokine storm and appropriate regulation of inflammatory response may be significant in the prevention and treatment of sepsis. This study evaluated the efficacy and safety of specific interleukin (IL)-1 inhibitors, specific IL-6 inhibitors, and GM-CSF blockades in the treatment of COVID-19 (at the edge of sepsis) patients through systematic review and meta-analysis. Methodology: A literature search was conducted on PubMed, EMBASE, Clinical Key, Cochrane Library, CNKI, and Wanfang Database using proper keywords such as “SARS-CoV-2,” “Corona Virus Disease 2019,” “COVID-19,” “anakinra,” “tocilizumab,” “siltuximab,” “sarilumab,” “mavrilimumab,” “lenzilumab,” and related words for publications released until August 22, 2021. Other available resources were also used to identify relevant articles. The present systematic review was performed based on PRISMA protocol. Results: Based on the inclusion and exclusion criteria, 43 articles were included in the final review. The meta-analysis results showed that tocilizumab could reduce the mortality of patients with COVID-19 (at the edge of sepsis) [randomized controlled trials, RCTs: odds ratio (OR) 0.71, 95%CI: 0.52–0.97, low-certainty evidence; non-RCTs: risk ratio (RR) 0.68, 95%CI: 0.55–0.84, very low-certainty evidence) as was anakinra (non-RCTs: RR 0.47, 95%CI: 0.34–0.66, very low-certainty evidence). Sarilumab might reduce the mortality of patients with COVID-19 (at the edge of sepsis), but there was no statistical significance (OR 0.65, 95%CI: 0.36–1.2, low-certainty evidence). For safety outcomes, whether tocilizumab had an impact on serious adverse events (SAEs) was very uncertain (RCTs: OR 0.87, 95%CI: 0.38–2.0, low-certainty evidence; non-RCTs 1.18, 95%CI: 0.83–1.68, very low-certainty evidence) as was on secondary infections (RCTs: OR 0.71, 95%CI: 0.06–8.75, low-certainty evidence; non-RCTs: RR 1.15, 95%CI: 0.89–1.49, very low-certainty evidence). Conclusions: This systematic review showed that tocilizumab, sarilumab, and anakinra could reduce the mortality of people with COVID-19 (at the edge of sepsis), and tocilizumab did not significantly affect SAEs and secondary infections. The current evidence of the studies on patients treated with siltuximab, mavrilimumab, and lenzilumab is insufficient. In order to establish evidence with stronger quality, high-quality studies are needed.

**Systematic Review Registration**: PROSPERO (https://www.crd.york.ac.uk/prospero/), identifier CRD42020226545

## 1 Introduction

Sepsis is a life-threatening organ dysfunction syndrome caused by host response imbalance due to an infection or infectious factors. The mortality and treatment expenditure of sepsis are relatively high, and there is no specific drug so far. An article published in *The Lancet* in 2020 pointed out that the number of sepsis patients worldwide reached 48.9 million in 2017, among which 11 million patients died, accounting for one-fifth of the global death toll (Rudd et al., 2020).

During the pandemic of coronavirus disease 2019 (COVID-19), patients with severe and critically ill COVID-19 may develop circulation disorders and severe lung damage. Some patients with multiple organ dysfunction, such as that of the liver and kidney, showed typical characteristics of sepsis and meet the diagnostic criteria for sepsis (Li et al., 2020). According to Sepsis-3, sepsis is defined as a life-threatening organ dysfunction caused by a dysregulated host response to infection. The organ dysfunction can be represented by an increase in the Sequential (Sepsis-Related) Organ Failure Assessment (SOFA) score of 2 points or more, which is associated with an in-hospital mortality greater than 10% (Singer et al., 2016). Recent studies have shown that patients with severe and critical diseases may experience immune hyperactivity with increased levels of interleukin (IL)-1, IL-6, granulocyte–monocyte colony-stimulating factor (GM-CSF), interferon-γ-inducible protein 10 (IP-10), tumor necrosis factor-α (TNF-α), and other several inflammatory cytokines and were associated with adverse clinical outcomes (Huang et al., 2020; Qin et al., 2020; Coomes and Haghbayan, 2020; Lucas et al., 2020). Therefore, inhibition of proinflammatory cytokines may be a potential therapeutic strategy in COVID-19 (at the edge of sepsis) patients. This study was the first to screen COVID-19 patients with sepsis or at the edge of sepsis through the SOFA score and systematically reviewed the efficacy and safety of anti-cytokine therapy, such as specific IL-1, IL-6 inhibitors, and anti-GM-CSF in COVID-19 patients with organ dysfunction (SOFA ≥2). This paper could help sepsis treatment strategy researchers to grasp the current status of anti-cytokine therapy for COVID-19 patients (at the edge of sepsis) and provide a new perspective for clinical treatment.

## 2 Methodology

This study was conducted in accordance with the Preferred Reporting Items for Systematic Reviews and Meta-analyses (PRISMA) guideline (Supplementary Material S1) (Moher et al., 2009) and registered with the National Institute for Health Research international prospective register of systematic reviews (PROSPERO registration number: CRD42020226545) (Wang et al., 2020).

### 2.1 Search Strategy and Selection Criteria

Electronic searches were carried out in PubMed, EMBASE, Clinical Key, Cochrane Library, China National Knowledge Infrastructure (CNKI), and Wanfang Database. The search terms that we used were “SARS-CoV-2,” “corona virus disease 2019,” “COVID-19,” “anakinra,” “tocilizumab,” “siltuximab,” “sarilumab,” “mavrilimumab,” and “lenzilumab” and relevant keywords for publications released until August 22, 2021. The search strategies are available as supplementary data (Supplementary Material S1). Other available resources were also used to identify relevant articles. The language will be limited to Chinese and English. Eligible articles were identified for inclusion by screening the titles, abstracts, and full text. Other relevant studies were manually screened by investigators from the reference list of included studies for further analysis. There was no date limit. Two independent reviewers (YW and KZ) carried out the search in a standardized process, followed with identifying eligible records through the examination of each title, abstract, and full text. Disagreements were resolved by consensus, and unresolved conflicts were decided by a third reviewer (QY).

The studies were selected based on the following inclusion criteria: (1) The patients were diagnosed with SARS-CoV-2 infection and their SOFA score (include mean value, median, and absolute value) ≥2 or, according to the SOFA scoring tool, a certain system index (including mean, median, and absolute value) should be within the range corresponding to the system score ≥2—for example, PaO_2_/FiO_2_ ratio (P/F) (including absolute value, mean value, or median value) was less than 300 mmHg (Singer et al., 2016). A SpO_2_/FiO_2_ ratio (S/F) of 315 corresponded with a P/F ratio of 300 mmHg [S/F = 64 + 0.84*(P/F)] (Rice et al., 2007). In this review, we defined such COVID-19 patients to be at the edge of sepsis. (2) The intervention of interest was a specific IL-1 inhibitor (anakinra), specific IL-6 inhibitors (tocilizumab, siltuximab, and sarilumab), GM-CSF blockades (mavrilimumab and lenzilumab) with or without standard of care (or treatment), and glucocorticoids. Comparator treatments included placebo, standard of care (or treatment), glucocorticoids, or no intervention; studies with no comparator group were also included. (3) Randomized clinical trials (RCTs), cohort studies, case–control studies, case series, case reports, clinical guidelines, protocols for clinical trials, and any other gray literatures will be included. The studies will not be limited in terms of country. The exclusion criteria were as follows: (1) The patients were not diagnosed as COVID-19; (2) The SOFA score (absolute value, mean value, or median value) of the patients was less than 2 or did not reach 2 on any of the system indicators; (3) Data on SOFA score or certain indicators in the SOFA scoring tool for the patients studied were not available in the study text, additional materials, or any other relevant resources; and (4) Studies without an available full text or whose data were incomplete or unavailable, posters, commentaries, letters, opinion articles, and *in vitro* studies were excluded. The defined primary outcome was all-cause mortality at 28–30 days. The safety outcomes included serious adverse events (SAEs) and (serious) secondary infection. Adverse events were graded according to the Common Terminology Criteria for Adverse Events, version 4.0 (National Institutes of Health, 2017).

### 2.2 Data Extraction and Quality (Risk of Bias) Assessment

Two independent reviewers (YW and KZ) extracted data from the eligible studies, and a third one (QY) validated it. The following information will be extracted: year of publication, authors, country, study type, sample size, participant demographics, time of administration, intervention characteristics (name of agent, dose, and route), concomitant medications, survival outcome, treatment-related adverse events, and conclusions of the authors.

The included studies were assessed in terms of potential bias by two reviewers (RD and RL) independently. The third researcher (XL) was consulted for resolving any difference of opinion. The Quality Assessment for Case Series of the National Institute for Health and Care Excellence will be used to evaluate the quality of the case reports (series). The total score is 8 points, in which a score of 4–8 is high quality, and a score less than 4 is low quality. The methodological quality for cohort and case–control studies was assessed based on the Newcastle–Ottawa Scale (NOS) (NOS, 2020). The total score is 9 points, in which scores of 0–3, 4–6, and 7–9 are respectively considered as low, moderate, and high quality. The methodological quality of RCTs was assessed based on the “Risk of Bias” 2.0 tool (Sterne et al., 2019). Each checklist item was judged as “low,” “moderate,” “serious,” and “critical,” The quality of evidence was assessed by using the “Grading of Recommendations Assessment Development and Evaluation (GRADE)” tool (Granholm et al., 2019). The quality of evidence of each outcome is classified as “high,” “moderate,” “low,” or “very low”.

### 2.3 Data Synthesis and Analysis

The Review Manager version 5.4.1 software was used for analyses. One reviewer (YW) would have to enter the data into the software, and another reviewer (M.L) would have to check the data for accuracy. For dichotomous outcomes, the number of events and total number of participants in the two groups were recorded. The different types of studies were analyzed separately, such as non-RCTs (cohorts and case–control studies) and RCTs. The risk ratio (RR) and odds ratio (OR) with 95% confidence intervals (CIs) were respectively assessed for non-RCTs and RCTs. Fixed-effects model was used if the result of the Q test was not significant (*p* > 0.1) and I^2^ <50%. Chi-square test, with a significance level at *p* ≤ 0.1, was used to assess the heterogeneity of treatment effects between trials. The *I* ^2^ statistic was used to quantify possible heterogeneity (75–100% considerable heterogeneity). We would explore potential causes through sensitivity and subgroup analyses if heterogeneity had been above 80%. We would not have conducted a meta-analysis if we had not found a reason for heterogeneity. If we could not perform a meta-analysis, we had planned to comment on the results from all studies.

## 3 Results

### 3.1 Search Results

**FIGURE 1:**
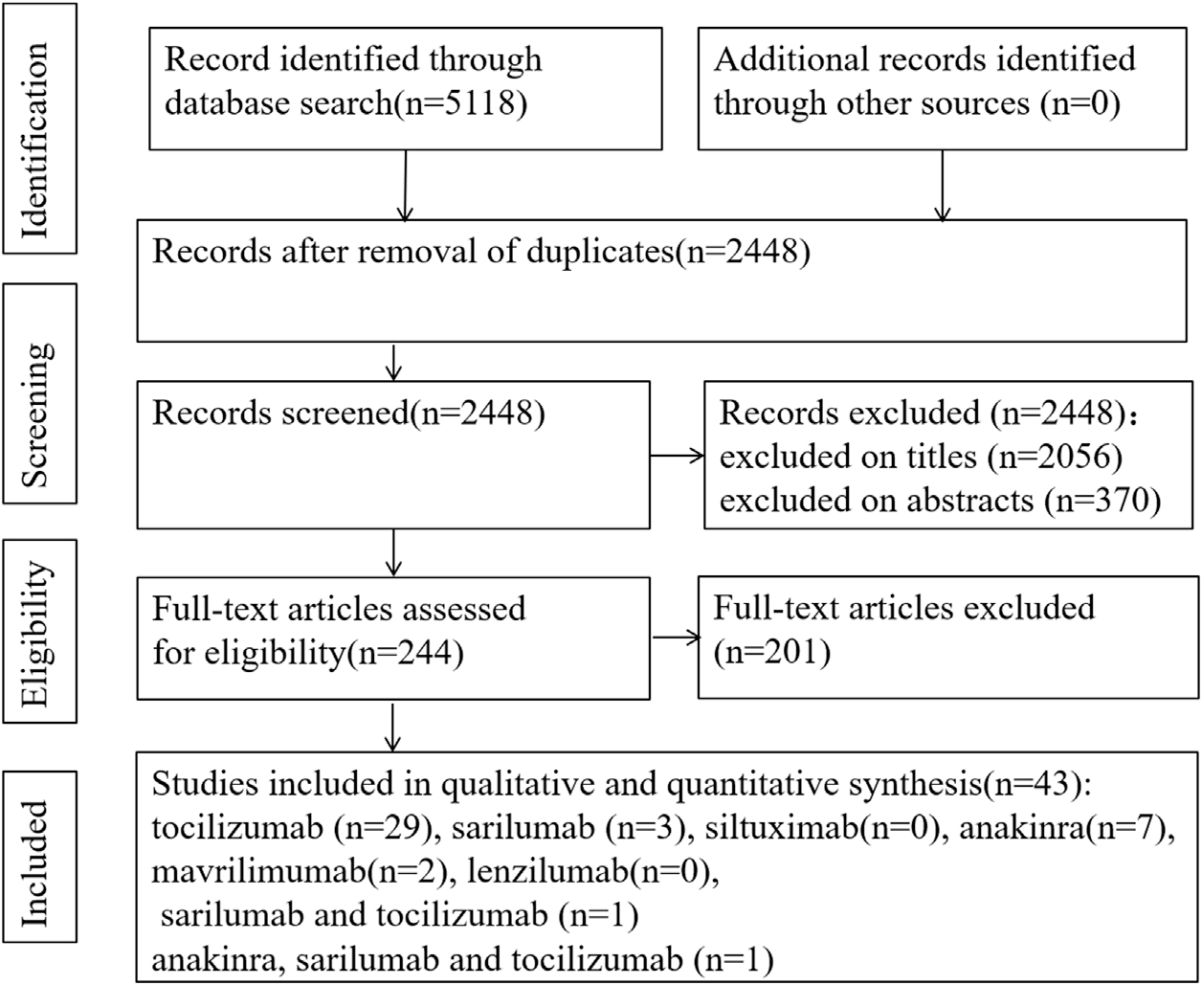
PRISMA flow chart of study selection.

Because of insufficient evidence available from RCTs, we also included cohort studies, case–control studies, and case reports (series). The search of the electronic databases on Aug 22, 2021 yielded a total of 5,118 studies. Following the elimination of duplicates and screening of titles and abstracts, we evaluated 244 articles in full text. Among these, we found 43 eligible articles (5 RCTs, 16 cohort studies, 2 case–control studies, and 20 case reports) (Figure 1) (Salvarani et al., 2021; REMAP-CAP Investigators et al., 2021; Canziani et al., 2020; Menzella et al., 2020; Fisher et al., 2021; Campochiaro et al., 2020; Vazquez Guillamet et al., 2021; Rajendram et al., 2021;Huang et al., 2021; Galván-Román et al., 2021; Saffo et al., 2021; Somers et al., 2021; Brosnahan et al., 2021; Corominas et al., 2021; Cavalli et al., 2021; Abe et al., 2021; Patel et al., 2020; Mady et al., 2020; Bernardo et al., 2020; Morillas et al., 2020; Cascella et al., 2020; Al-Kaf et al., 2021; Eroglu et al., 2021; Kataoka et al., 2021; Kishaba et al., 2021; Leelayuwatanakul et al., 2021; McKenzie et al., 2021; Nourié et al., 2021; Ladna et al., 2021; Senegaglia et al., 2021; Thammathiwat et al., 2021; Lescure et al., 2021; Della-Torre et al., 2020; Gremese et al., 2020; Kyriazopoulou et al., 2021a; Bozzi et al., 2021; Franzetti et al., 2021; Kyriazopoulou et al., 2021b; Erden et al., 2021; Filocamo et al., 2020; Franzetti et al., 2020; Cremer et al., 2021; De Luca et al., 2020). All studies were published in peer-reviewed journals.

In the process of full-text review, there were four articles for which we failed to obtain the full texts. The four studies were related to tocilizumab. Two studies did not report the efficacy and safety of tocilizumab (Garg et al., 2020; Kashin et al., 2020). The other two studies were case reports, in which one patient developed tuberculosis reactivation during treatment and the other patient had a secondary infection. The authors of the two case reports suggested that patients might be at a high risk for secondary infection after receiving tocilizumab or tocilizumab combined with glucocorticoid. They suggested that clinicians should use tocilizumab with caution and screen for latent tuberculosis before medication (Mazankova et al., 2020; Moideen et al., 2020).

### 3.2 Risk of Bias Assessment

The risk of bias of the RCTs was low to moderate, respectively. The results are shown in Supplementary Figure S1 (Supplementary Material S2, Appendix p1). Some studies reported only one outcome, and we assessed the risk of bias for the results—for instance, bias in the measurement of outcomes was not available for safety for the study of Brosnahan *et al*. (2021) because they did not report it. For mortality outcomes, the methodological quality of 16 cohorts was moderate to high, and those of 2 case–control studies were moderate. For safety outcomes, the methodological quality of 14 cohorts was low to high, and those of 2 case–control studies were low to moderate (NOS assessment results are shown in Supplementary Material S2, Appendix p2–40). The methodological quality evaluation results of the included case reports (series) showed that the quality was low to moderate (the results of quality are shown in Supplementary Material S2, Appendix, p41–42).

### 3.3 Characteristics of Patients

The 43 studies included were identified and critically evaluated, which included a total of 4,951 patients with confirmed SARS-CoV-2 infection, of whom 2,243 received mechanical ventilation. Only 11 studies reported the SOFA score of enrolled patients, of which 4 studies reported SOFA scores greater than or equal to 6 (tocilizumab), 3 studies reported scores between 4 and 5 (tocilizumab), and 4 studies reported scores between 2 and 3 (3 for anakinra, 1 for mavrilimumab). The remaining 32 articles reported the respiratory status (including P/F or S/F) and platelet of patients, of which 5 studies included patients with P/F less than or equal to 100 mmHg and of which 13 studies reported patients with P/F between 100 and 200 mmHg.

Most patients received standard of care (or standard of treatment) based on local treatment guidelines. However, the medication regimens of the standard of care were different, mainly including antiviral drugs, antibiotics, glucocorticoids, and other symptomatic drugs. Anti-cytokine agents were mainly used by intravenous injection and, in a few studies, by subcutaneous administration. In addition, there is still no consensus on the dosage of anti-cytokine agents for such patients until now. In the included articles, the dosage of most patients was as follows: tocilizumab, 8 mg/kg/dose and up to a maximum of 800 mg; sarilumab, 400 mg/dose with 1 to 2 doses; anakinra 100 mg/dose 1–4 times a day; and mavrilimumab 6 mg/kg/dose. The characteristics of the included studies are presented in Table 1.

**TABLE 1:** Characteristics of the included studies.

Study	Country	Study type	P	SOFA score or indicators, median (IQR)	Laboratory values, median (IQR)	Intervention group	Control group	Time of administration, median (IQR)-days	Dosage	Usage	Comorbidities(n)	Concomitant medications(n)	Effective outcomes	Safety outcomes	Authors’ conclusion
Salvarani et al. (2020)	Italy	RCT, MC, open-labal	126; 77 M:49 F	P/F: 264.5 (243.0–290.0) mmHg	CRP- mg/dl: 8.2 (3.7–13.5).; IL-6-pg/ml: 42.1 (20.6–74.9); ferritin-ng/ml: 569.0 (317.0–1,156.0)	TCB + standard of care	Standard of care	Days from symptom onset to randomization, median (IQR): 8.0 (6.0–11.0)	A dose of 8 mg/kg up to a maximum of 800 mg, followed by a second dose after 12 h	Intravenous	DB (19), obesity: BMI ≥30 (38), HTN (56), COPD (4)	HCQ (115), antiretrovirals (52), azithromycin (26), steroids	Within 14 days, 17 of 60 in the experimental group and 17 of 63 in the control group showed clinical worsening; 1 in the control group and 2 patients in the experimental group died before 30 days from randomization	AE (*n*, %): TCB (14, 23.3%); control (7, 11.1%). SAE (*n*): TCB (1), control group (2)	No benefit on disease progression was observed compared with standard care
REMAP-CAP Investigators, et al. (2021)	United Kingdom, Australia, Canada, New Zealand, France	RCT, MC, open label	865; 629 M:236 F	P/F: 116.5 (89–165) mmHg	CRP-µg/ml: 136 (79–208)	TCB, sarilumab	Standard of care	Median days from hospital admission to enrollment (IQR): 1.2 (0.8–2.8)	TCB: 8 mg/kg (up to a maximum of 800 mg), 1 to 2 doses; sarilumab: 400 mg, 1 dose	Intravenous	DB (304), Kidney disease (81), respirator (206)	COVID-19 antivirals, corticosteroid	The analysis of 90-day survival showed that the survival rate of the IL-6 inhibitor groups was improved. Compared with the control group, the HR was 1.61 (95%CI 1.25–2.08) and the posterior probability of superiority was more than 99.9%	SAEs: 9 in the TCB group, 11 in the control group, and 0 in the sarilumab group	In critical patients with organ support, treatment with TCB and sarilumab improved the outcomes
Canziani et al. (2020)	Italy	MC, retrospective cohort	128; 94 M:34 F	P/F, mean (SD): 94 (67) mmHg	Ferritin, mean (SD)-ng/ml:1,604 (1,201); CRP, mean (SD)-g/dl: 19.1 (8.6)	TCB + standard of care	Standard of care	Time since symptom onset (SD): 11 (6)	8 mg/kg per dose, followed by a second dose 24 h later if no clinical worsening occurred	Intravenous	CCI, mean (SD): 2.4 (1.6); HTN (66)	HCQ, azithromycin, glucocorticoids	TCB did not significantly affect the risk of death, but TCB was associated with the use at baseline of NIV or invasive MV and the presence of comorbidities	TCB was not associated with the risk of infections, bleeding, or thrombosis	TCB did not affect the 30-day mortality in severe respiratory impairment patients
Menzella et al. (2020)	Italy	SC, retrospective, case–control study	79; 56 M:23 F	SOFA score: mean (SD): 4.3 (1.3)	CRP, mean (SD)-mg/dl: 11.9 (7.2); IL-6, mean (SD)-pg/m: 147.2 (180.4)	TCBb + standard therapy	Standard therapy	–	Intravenous: 8 mg/kg—max 800 mg, Q12 h; subcutaneous: 2 to 4 doses of 162 mg	Intravenous	Number of comorbidities, mean (SD): 2.9 (2.1)	HCQ (20), azithromycin (60), methylprednisolone	The probability of death and intubation in patients treated with TCB was significantly lower than that in patients not treated with TCB	2 patients treated with TCB developed cavitating lung lesions	TCB may be helpful in COVID-19 patients with severe respiratory impairment receiving NIV
Fisher et al. (2021)	United States	SC, retrospective cohort study	115; 80 M:35 F	SOFA score within 24-h intubation: 6.0 (3.0)	CRP-mg/dl: TCB 19.5 (15.7); control 17.6 (18.0); ferritin-ng/ml: TCB 1,507 (1,518), control 1,462 (1,435)	TCB + standard of care	Standard of care	Mean time from intubation to treatment was 2.5 days	400 mg/dose	Intravenous	CCI: TCB 2.0 (3.0), control 3.0 (3.0)	HCQ (108), corticosteroids	No reduction in mortality associated with receipt of TCB	The increased risk of secondary infection in patients given TCB was not observed	TCB was not associated with a reduction in mortality in MV patients with COVID-19 after controlling for severity of illness, age, and co-morbidities
Campochiaroa et al. (2020)	Italy	SC, retrospective, cohort	65; 56 M:9 F	P/F-mmHg: TCB 107 (82–181), control 124 (91–172)	CRP-mg/L: TCB 156 (100–208); control: 169 (98–226); ferritin-ng/ml: TCB 1,400 (1,027–2,777); 1,448 (793—4,131)	TCB + standard treatment	Standard treatment	Duration of symptoms (days): TCB11 (8–14); control: 9 (8–10)	400 mg/dose; in case of respiratory worsening, a second dose was given after 24 h	Intravenous	HTN (28), CKD (8), DB (10), CAD (10)	HCQ, azithromycin	During the 28-day follow-up, mortality was 33% in the standard treatment group and 33% in the TCB group	The rate of pulmonary thrombosis and infection was similar between the two groups	At day 28, compared to standard treatment, the clinical improvement and mortality were not statistically different
Vazquez Guillamet et al. (2021)	United States	SC, retrospective cohort study	43; 16 M:27 F	P/F-mmHg: 171.5 (122–221)	CRP- mg/dl:142.7 (97.7–213.7); IL-6-pg/ml: 61 (28.6–439)	TCB + standard care	Standard care	–	8 mg/kg, a second dose at 12–24 h later	Intravenous	DB (12), CKD (17), CAD (11); Charlson score, median (IQR): TCB 0 (0–3), control 2 (0–4)	——	Treatment with TCB was a significant predictor of survival	Treatment with TCB might increase the risk of infection	After adjusting for severity of critical illness, administration of IL-6 inhibitor was associated with improved survival
Rajendram et al. (2021)	United States	MC, retrospective observational cohort	164; 103 M:61 F	SOFA score, mean (SD): TCB 6.0 (3.3), control 6.4 (3.6); P/F, mean (SD)-mmHg: TCB 134.8 (68.4), control 149.8 (82.2)	CRP, mean (SD)-mg/dl: TCB 20.4 (10.1), control 17.2 (12.3); ferritin- ng/ml: TCB 1,398.2 (1,143.3), control 4,159.9 (13,454.1)	TCB	Did not receive TCB	–	4–8 mg/kg (maximum dose 400 mg), one dose only	Intravenous	DB (67), COPD (48)	HCQ (88), azithromycin (78), systemic corticosteroids	ICU mortality was lower in the TCB group, with more hospital-, ICU-, and vasoactive-free days at day 28 compared with those who did not receive TCB. There was no difference in MV-free days at day 28 or development of secondary infections	There was no difference in the rates of secondary infection	Administration of TCB might decrease in ICU mortality of critical COVID-19 patients with severe hypoxemic respiratory failure
															TCB use was associated with a significant decrease in ICU death rate in critically ill COVID-19 patients with severe hypoxemic respiratory failure
Huang et al. (2021)	United States	SC, retrospective cohort study	96; 64 M:32 F	SOFA score median (range): TCB 4 (0–13), non-TCB 5 (0–13)	CRP, median (range)-mg/L: TCB 122.3 (2.4–327.2); non-TCB 122.5 (11.10–343.1)	TCB	Non-TCB	Median (range): TCB:2 (0–16)	A single 400 mg dose	Intravenous	HTN (62), cardiac arrhythmias (33), DB (47), other comorbidities (84)	HCQ (63), azithromycin (69), and remdesivir (9), DXMS	Fewer deaths were observed among TCB-treated patients, both in the overall population and among the subgroup of patients requiring MV	Secondary infections were not different between the two groups and were predominantly related to invasive devices, such as urinary and central venous catheters	Administration of TCB was associated with fewer deaths compared to non-treatment despite predominantly being used in patients with more advanced respiratory disease
Galván-Román et al. (2021)	Spain	SC, retrospective observational study	146; 97 M:49 F	Baseline P/F, median (p25–p75): 215 (112–310)	CRP, median (p25–p75)-mg/dl: 11.55 (5.16–22.53)	TCB	Not treated with TCB	Duration of symptoms at admission, median (p25–p75)-d: 6 (4–7)	8 mg/kg (maximum 800 mg) followed by a second one after 12 h	Intravenous	Comorbidities, median (p25–p75): 100 (69)	HCQ (136), azithromycin (82), Lpv/Rtv(119), and glucocorticoids	Early TCB treatment might improve in oxygenation (P/F) in patients with high IL-6. Patients with high IL-6 not treated with TCB showed a high mortality as well as those with low IL-6 treated with TCB	Relevant SAEs were not observed in TCB-treated patients	Baseline IL-6 more than 30 pg/ml predicts IMV requirement in patients with COVID-19 and contributes to the establishment of an adequate indication for the treatment of TCB
Saffo et al. (2021)	United States	Retrospective cohort	130; 93 M:37 F	SOFA score mean (SD): TCB 5.7 (2.2); control 6.0 (3.2)	Mean (SD) IL-6-pg/ml: TCB 108.8 (179), control 62.3 (105.3); mean (SD) CRP-mg/dl: TCB 13.2 (7.4); control: 12.6 (6.4)	TCB	Did not receive TCB	Mean (SD) symptom onset to admission: TCB 6.9 (3.4); control 7.1 (4.4)	A dose of 8 mg/kg; 400 mg single dose	Intravenous	HTN (98), DB (53), COPD (27), CA (24)	HCQ, azithromycin, methylprednisolone	A Kaplan–Meier survival curve demonstrated no difference in survival between TCB and comparator patients. In the multivariable Cox regression model for mortality at 30 days, administration of TCB was not associated with decreased mortality	Positive blood culture was not statistically significant between the groups	No difference in survival was observed in critical patients treated with TCB
Somers et al. (2021)	United States	SC, observational controlled cohort	154; 102 M:52 F	P/F (*n* = 80): 165 (136.5–231.5) mmHg	CRP-mg/L: 220 (125–293); ferritin-ng/ml:1,418 (692–2,139)	TCB	Did not receive TCB	–	8 mg/kg (maximum 800 mg) × 1 dose	Intravenous	HTN (102), DB (25), CKD (64), asthma (31)	HCQ, remdesivir, corticosteroid	In IPTW-adjusted models, TCB was associated with a 45% reduction in hazard of death	TCB was associated with an increased proportion of patients with superinfections, but there was no difference in the 28-day case fatality rate between the two groups	In the cohort, administration of TCB was associated with a lower mortality in spite of higher superinfection occurrence
Brosnahan et al. (2021)	United States	MC, retrospective cohort	537; 366 M:171 F	SOFA score: TCB:7 (7–9); control: 8 (3–10)	——	Steroid + TCB	Steroid	–	A total of 90% of patients received 400 mg as a single dose	Intravenous	HTN without complication (394); DB without complication (229)	Antivirals, immune globulins, HCQ, methylprednisolone, DXMS, hydrocortisone, prednisone	The combination group (TCB 400 mg and daily equivalent DXMS 10 mg) had an improved 28-day mortality compared with the steroid-only group (daily equivalent DXMS 10 mg) without increasing the risk of infection	There was no statistical significance difference in the rate of infections between the propensity-matched groups	The combination group had an improved 28-day mortality compared with the steroid-only group without increasing the risk of infection
Corominas et al. (2021)	Spain	Single-center, observational study	104; 72 M:32 F	P/F, mean (SD): 201.3 (78.1) mmHg	IL-6-pg/ml, mean (SD): 171.6 (40–210.7); CRP-mg/L, mean (SD): 198.4 (161.5)	TCB		–	If ≥75 kg: a single dose of 600 mg, less than <75 kg: a single dose of 400 mg	–	HTN, dyslipidemia, obesity, CLD, DB	HCQ	The overall mortality rate was 5.8% patients. Mortality in hospitalized non-TCB treated patients was 10%. The regional death rate was 11%	–	Early treatment of IL-6 inhibition in COVID-19 patients with imminent hyper-inflammatory response may be safe and effective
Cavalli et al. (2021)	Italy	Cohort study	392; 301 M:91 F	P/F ≤ 300 mmHg	CRP-mg/L: 129 (100–171)	Anakinra, tocilizumab, sarilumab	No interleukin inhibitors	None	Anakinra: 5 mg/kg/dose twice daily (total daily dose of 10 mg/kg) until clinical benefit. TCB: 400 mg/dose, which was repeated after 24 h if the respiratory function further worsened; sarilumab: 400 mg/dose	Intravenous	CAD (119); history of neoplasia (66); DB (72)	HCQ, glucocorticoid	There was no difference in adverse clinical outcome risk in patients treated with IL-6 or IL-1 inhibition relative to patients who did not receive interleukin inhibitors	–	IL-1 inhibition was associated with a significant reduction of mortality in COVID-19 patients. IL-6 and IL-1 inhibition were effective in patients with low lactate dehydrogenase concentrations
Abe et al. (2020)	Japan	Case reports	2; 1 M:1 F	PLT <20*10^9^/L	P1: IL-6-pg/ml: 47.8; P2: IL-6-pg/ml: 93.6	TCB		Days from symptom onset to TCB application: P1: 8 days later; P2: 4 days later	8 mg/kg of TCB twice	Intravenous	DB; KD	P1: peramivir and favipiravir; P2: peramivir and favipiravir, immunoglobulin	P1: He was released from the isolation unit on day 29. P2: She was released from the isolation unit on day 36 based on negative results of PCR assays	–	Anti-cytokine therapy might be effective for severe COVID-19 in end-stage renal disease patients
Patel et al. (2020)	United States	Case reports	1; F (12 years old)	PLT <10*10^9^/L	IL-6-pg/ml: 34; CRP-mg/dl: 8.3	TCB		Days from symptoms onset to TCB application: 12	2 doses of TCB (8 mg/kg 12 h apart)	Intravenous	Severe thrombocytopenia	HCQ, remdesivir, immunoglobulin; methylprednisolone	Discharged	–	Treatment with cytokine-directed agents such as TCB could be considered in critical patients
Mady et al. (2020)	Saudi Arabia	A case series	61; 54 M:7 F	SpO_2_/FiO_2_, median (IQR): 162 (145–209.2) mmHg	CRP, median (IQR)-mg/L: 31.7 (30.5–49.9)	TCB		–	8 mg/kg (two consecutive intravenous infusions 12 h apart)	Intravenous	More than one comorbidity (%): 38 (62.3%)	Lpv/Rtv or ribavirin	Administration of TCB did not affect the mortality of critical COVID-19 patients	No SAEs due to TCB were recorded; 12 patients developed nosocomial acquired infections	TCB could be an adjunct safe therapy in rapidly evolving COVID-19 and associated critical illness
Bernardo et al. (2020)	Italy	Case reports	1; M	P/F: 295 mmHg	CRP-mg/L: 89.8 (9 × the upper limit of normal)	TCB		8th day of admission	Two doses of TCB 8 mg/kg administered 12 h apart	Intravenous	Hypertensive cardiomyopathy, paroxystic AF, CRD	HCQ, Lpv/Rtv	On day 30 after the TCB injections, the ANC of the patient began to improve in spite of far-from-normal values	Severe prolonged neutropenia	Considering the increasing use of TCB in COVID-19 patients, this case warrants further studies regarding the possible adverse hematological effects that need to be monitored in order to prevent superimposed infections
Morillas et al. (2020)	United States	Case reports	5; 2M:3F	P1: P/F: 196 mmHg; P2: P/F: 200 mmHg; P3: P/F: 113 mmHg; P4: P/F: 283 mmHg; P5: P/F: 156 mmHg	P1: CRP-mg/dl: 9.7, IL-6-pg/ml: 7; P2: CRP-mg/dl: 18.8, IL-6-pg/ml: 13; P3: CRP-mg/dl 36.5, IL-6-pg/ml: 438; P4: CRP-mg/dl: 5.2; P5: CRP-mg/dl: 32.7	TCB		TCB administration after symptoms onset (day): P1: 7; P2: 5; P3: 10; P4: 13; P5: 12	P1: 400 mg/dose; P2: 400 mg/dose; P3: 400 mg/dose; P4: 310 mg/dose (adjusted based on weight); P5: 400 mg/dose	Intravenous	P1: HTN, kidney transplants; P2: lung transplant, DB, HF, HTN, hemodialysis, CA; P3: a motor vehicle accident on tacrolimus; P4: liver transplant, rheumatic heart disease, CKD stage 3; P5: HTN, DB, pulmonary embolism, coronary artery by-pass graft surgery, and kidney transplantation	P1: Lpv/Rtv; P2/3: HCQ, steroids	P1/2/3/5: discharged; P4: Ddied	There were no immediate drug-related side effects, although 2 patients developed 3 proven bacterial infections within 14 days after dosing	TCB can be used without major direct toxicity in SOT/CTTRs early after initiation of MV due to COVID-19, regardless of type of organ transplanted
Cascella et al. (2020)	Italy	Case reports	1; M	P/F: 150 mmHg	CRP-mg/L: 193; IL-6-pg/ml: 93	TCB		2nd day of admission	8 mg/kg, 800 mg	Intravenous	–	Lpv/Rtv, HCQ	Discharged	–	The combination of IL-6 inhibitor with calibrated ventilatory strategies may improve outcomes
AL-Kaf et al. (2021)	Kingdom of Saudi Arabia	Case reports	1; M	P/F: 133 mmHg	IL-6-pg/ml: 130	TCB		5th day of admission	–	–	Down syndrome	Hydrocortisone	After a total of 31 days of MV support, the patient was successfully weaned off and planned for tracheostomy closure	–	This is a rare critical presentation of COVID-19 in a Down syndrome patient with cardiac tamponade, ARDS, and severe hypothyroidism who responds well to pericardiocentesis, levothyroxine, hydrocortisone and TCB
Eroglu et al. (2021)	Turkey	Case reports	1; M	P/F: 204 mmHg	IL-6-pg/ml: 14 (0–6.4); CRP-mg/L: 78 (0–5)	TCB		8th day of admission	TCB 400 mg was infused 2×/day for 2 days	Infused intravenously	HTN	Favipiravir, oseltamivir, HCQ	On day 14, the patient was transferred to a negative COVID-19 service with negative PCR test and better clinical condition	–	Patients with severe COVID-19 should be monitored for hemophagocytic lympho-histiocytosis syndrome, and TCB can be used early under NIV delivered by helmet mask
Kataoka et al. (2021)	Japan	Case reports	1; M (age 85)	P/F: 100 mmHg	CRP-mg/dl: 13.02; IL-6-pg/ml: 154	TCB		–	A single dose of 480 mg/body	Intravenous	Sjögren’s syndrome	Favipiravir, ciclesonide	Discharged	–	IL-6 inhibition may be an optional treatment in patients with a severe respiratory condition
Kishaba et al. (2021)	Japan	Case reports	1; M (age 74)	P/F: 115 mmHg	CRP-mg/dl: 32	TCB		On the day of admission	480 mg (8 mg/kg/day)	Intravenous	HTN	Favipiravir, methylprednisolone	Discharged	–	In order to secure good outcomes in critical COVID-19 patients, early administration of intensive combination therapy requires suppression of both viral replication and inflammation
Leelayuwatanakul et al. (2021)	Thailand	Case reports	2; 1 M:1 F	P1: P/F: 260 mmHg; P2: P/F: 130 mmHg	P1: CRP-mg/L 228; IL-6-pg/ml: 1,091; P2: hs-CRP-mg/L:225; IL-6-pg/ml: 426.2	TCB		P1: 8th day of admission; P2: 4th day of admission	400 mg/dose	Intravenous	P1: HIV infection, DB, dyslipidaemia; P2: relapse multiple myeloma, triple vessel disease, DB, HTN	Favipiravir, HCQ, darunavir/ritonavir, immunoglobulin	Discharged	–	Combined therapeutic modalities are promising treatment for severe COVID-19 infection in the immunocompromised host. Timely adjunctive therapies that alleviate overwhelming inflammation may improve the outcome
McKenzie et al. (2021)	United States	Case series	16; 9M:7 F	P/F: 84 (IQR: 69–108.6) mmHg	CRP-mmol/L: 219.6 ± 72.1; IL-6-pg/ml: 248.7 (IQR: 69.5–719.2)	TCB		Time from admission to administration: 7.08 ± 3.5 (days)	400 mg (400–600)	Intravenous	HTN (31%); DB (25%)	HCQ, convalescent plasma, steroids	8 (50%) patients were discharged home, 7 (44%) patients died, and 1 (6%) patient was still hospitalized at the end of data collection	–	The study did not support the effectiveness of TCB in the treatment of critical COVID-19 patients
Nourié et al. (2021)	France	Case reports	1; M	SOFA score: 13	CRP-mg/L: 62.2	TCB		On day 7 of the illness	400 mg/dose	–	Von Hippel–Lindau; HTN; DB; CAD	HCQ, hydrocortisone	Discharged	–	A single dose of 400 mg of TCB was effective and well tolerated
Ladna et al. (2021)	United States	Case series	2; 2 M	P1: P/F: 117 mmHg; P2: P/F: 116 mmHg	P1: IL-6-pg/ml 45; hs-CRP-mg/L: 74.9; P2: IL-6 pg/ml:31; hs-CRP-mg/L: 244	TCB		First hospital day	400 mg/dose	–	P1: CAD, DB, HTN, and prior stroke; kidney and heart transplant; P2: chronic hepatitis B complicated by hepatocellular carcinoma status post orthotopic liver transplant, HTN, DB	HCQ, P1: tacrolimus, hydrocortisone, mycophenolate; P2: hydrocortisone	Discharged	–	TCB appears to hold promise for critical COVID-19 patients who require MV when given shortly after intubation
Senegaglia et al. (2021)	Brazil	Case report	1; M	P/F: 87 mmHg	CRP-mg/ml: 25.3 (<5.0)	TCB		2nd, 3rd hospital day	400 mg/dose for 2 days	Infusion	HTN, urethral stenosis	DXMS	Discharged	No adverse events	Administration of TCB and mesenchymal stromal cells proved to be safe, and the results of the report prove to be a promising alternative in patients with severe acute respiratory syndrome
Thammathiwat et al. (2021)	Thailand	Case report	1; M	P/F: 226 mmHg	IL-6-pg/ml: 17.1 (reference level <7)	TCB		6th hospital day	8 mg/kg/dose	–	Kidney transplantation, HTN, dyslipidemia, and post-transplant DB	Darunavir, ritonavir, favipiravir, tacrolimus, prednisolone, and immunoglobulin	COVID-19 was undetected	–	For this COVID-19 patient with kidney transplant, favipiravir together with decreased immunosuppression and IL-6 inhibitor antibody provides favorable outcomes. Decision on timing for IL-6 inhibitor initiation can be guided by IL-6 level monitoring
Lescure et al. (2021)	Argentina, Brazil, Canada, Chile, France, Germany, Israel, Italy, Japan, Russia, and Spain	MC, RCT	416, 261 M:155 F	SpO_2_/FiO_2_, median (IQR)-mmHg: 237.5 (173.6–300.0)	CRP-mg/L: 94.6 (48.1–167.9); IL-6-pg/ml: 12.3 (4.8–25.5)	Sarilumab	Placebo	Time from dyspnea onset to baseline, days: 5.0 (2.0–9.0)	200 mg, 400 mg	Intravenous	HTN (177), DB (110), CA (42), obesity (86), HL (41), CAD (22), COPD (18)	HCQ/CQ, azithromycin, remdesivir, convalescent plasma, and corticosteroids	At day 29, there were numerical, non-significant survival differences between sarilumab 400 mg and placebo for critical COVID-19 patients	No unexpected safety signals were seen	The result of this study did not show the efficacy of sarilumab in patients admitted to the hospital with COVID-19 and receiving supplemental oxygen
Della- Torre et al. (2020)	Italy	SC, open- label cohort study	56; 44 M:12 F	P/F <300 mmHg	CRP-mg/L: 152 (116–210); ferritin-ng/ml: 1,376 (1,023–6,927)	Sarilumab + standard care	Standard of care	Duration of symptoms before enrollment (days): 7 (7–10)	400 mg	Intravenous	HTN (17), DB (9), HLD (8), COPD (2), and CAD (6)	HCQ, Lpv/Rtv, azithromycin, corticosteroids	A total of 61% of patients treated with sarilumab experienced clinical improvement and 7% of patients died. These findings were not significantly different from the comparison group (clinical improvement 64%, mortality 18%)	The rate of pulmonary thrombosis and infection was similar between the 2 groups	Overall mortality and clinical improvement were not significantly different between standard of care and sarilumab. Sarilumab was associated with faster recovery in a subset of patients showing minor lung consolidation at baseline
Gremese et al. (2020)	Italy	SC, observational cohort	53; 47 M:6 F	P/F-mmHg: medical wards 146 (120–212), ICU 112 (100–141.5)	–	Sarilumab + standard care	No control group	–	400 mg, 1 to 2 doses	Intravenous	34 (64.2%) had at least one comorbidity	Darunavir/ritonavir; Lpv/Rtv	Within the medical wards, 89.7% of inpatients significantly improved, 70.6% were discharged. Within the ICU, 64.2% were discharged from ICU to the ward and 35.8% were still alive at the last follow-up	Sarilumab appears to be safe	IL-6 receptor inhibition leads to good clinical outcome in severe COVID-19 patients, and sarilumab is a safe and effective alternative in the therapeutic armamentarium of this disease without a defined standardized treatment
Kyriazopoulou et al. (2021a)	Greece, Italy	MC, RCT	594; 344 M:250 F	SOFA score, mean (SD): 2.4 (1.1); P/F-mmHg: 237 (181–301)	CRP, mean (SD)-mg/L: 50.6 (25.3–99.7); IL-6, mean (SD)-pg/ml: 16.8 (7.0–39.8); ferritin, mean (SD)-ng/ml: 585.2 (294.5–1,047.0)	Anakinra + standard of care	Placebo + standard of care	From symptom onset to start of study drug (days), median (Q1–Q3) 9 (7–11)	100 mg/daily,7–10 days	Subcutaneous	CCI, mean (SD): 2.2 (1.6); DB (94), chronic heart failure (18), CRD (10), COPD (24), CAD (41), Atrial fibrillation (28), depression (34)	Remdesivir, DXMS	Anakinra protected from severe disease or death; the 28-day mortality decreased	The incidence of treatment-emergent SAEs through day 28 was lower in patients in the anakinra combined with standard of care group compared to the placebo combined with standard of care group	Early administration of anakinra guided by suPAR shows 2.78 times better improvement of overall clinical status in moderate and severe COVID-19
Bozzi et al. (2020)	Italy	SC, prospective observational cohort	120; 96 M:24 F	P/F-mHg: 151 (105–204.5), 32.5% patients on MV	Ferritin-mcg/L: 1,555 (1,239–2,679); CRP-mg/dl: 15.2 (10.8–23.1)	Anakinra + methylprednisolone + standard treatment	Methylprednisolone + standard treatment	Days between hospitalization and inclusion: intervention: 3 (1–6); control: 1 (0–2)	200 mg q8 h for 3 days and then 100 mg q8h up to day 14	Intravenous	Median CCI was 0 (IQR 0–1)	HCQ, Lpv/Rtv, remdesivir, methylprednisolone	At 28 days, mortality was 35.6% in controls and 13.9% in treated patients. Unadjusted and adjusted risk of death was significantly lower for treated patients compared to controls	No significant differences in laboratory alterations or bloodstream infections were observed	Administration of anakinra combined with methylprednisolone may be an effective therapy in COVID-19 patients with respiratory failure and hyper-inflammation, also on MV
Franzetti et al. (2021)	Italy	MC, retrospective cohort study	112; 87 M:25 F	P/F: 133 (110–196) mmHg	CRP-mg/dl: 17.5 (11.0–24.9); ferritin-ng/ml: 1,620 (918–2,988)	Anakinra + standard of care	Standard of care	Symptom duration before hospitalization: 7 (5–10)	Regular ward: 7 days at a dosage of 100 mg four times a day, subcutaneous; ICU: 200 mg three times daily, intravenous	Intravenous or subcutaneous	CCI median (IQR): 3 (2–4); HTN (59), ischemic heart disease (20), COPD (8), DB (19)	HCQ, Lpv/Rtv	Survival at day 28 was significantly higher in anakinra-treated patients than in the controls. When stratified by continuous positive airway pressure support at baseline, the survival of anakinra-treated patients was also significant compared with the controls	No significant difference was observed in the rate of infectious-related adverse events between groups	Anakinra improved the invasive ventilation-free survival and overall survival and was well tolerated in patients with ARDS associated with COVID-19
Kyriazopoulou et al. (2021b)	Greece	MC, cohort	260; 165 M:95 F	SOFA score: 2 (1); P/F mmHg: anakinra 293.3 (195.7–371.2), parallel SOC after propensity matching 285.7 (208.5–371.7)	CRP-mg/L: anakinra 47.4 (14.3–105.5), parallel SOC after propensity matching 68.8 (19.7–141.8)	Anakinra + SOC	SOC	Days from onset of symptoms to start of treatment, median (range): anakinra 8 (1–23), parallel SOC after propensity matching 7 (1–12)	100 mg once daily for 10 days	Subcutaneous	CCI, mean (SD): 3 (2); DB (73), CAD (21), CRD (5), HTN (125), COPD (18)	HCQ, remdesivir, azithromycin, DXMS	22.3% with anakinra treatment and 59.2% comparators progressed into severe respiratory failure; the 30-day mortality was 11.5 and 22.3%, respectively	The rate of SAEs was lower among anakinra-treated patients	Early soluble urokinase plasminogen activator receptor guided anakinra decreased severe respiratory failure and restored the pro-/anti-inflammatory balance
Erden et al. (2021)	Turkey	SC, retrospective case review	17; 12 M:5 F	SOFA score, median IQR: 3 (3)	CRP-mg/L: 45.6 (101.8); ferritin concentration-μg/L: 397 (307)	Anakinra	No control group	Duration of COVID-19 symptoms before anakinra, days: 7 (4.5)	100 mg/12 h from day (D) 1 to D3, then at 100 mg/24 h from D3 to D5	Subcutaneously	HTN (9), DB (4), asthma (1), CAD (3), CA (1)	HCQ, azithromycin, favipiravir	The mortality rate was 17.6%; 1 patient was receiving low-flow oxygen supply, 3 patients no longer needed oxygen supply, and 10 patients were discharged	Treatment was well tolerated	The other factors that enhance the administration of anakinra in the situation of viremia could also be sorted as no response to full-dose antiviral drugs, antiviral side effects, or no success to antiviral treatment
Filocamo et al. (2020)	Italy	Case report	1; M	P/F:160 mmHg	–	Anakinra		Day 10 after admission	200 mg intravenously followed by 100 mg/6 h subcutaneously	Intravenous and subcutaneous	–	Lpv/Rtv, HCQ	Discharged	–	This critical COVID-19 patient was successfully treated with IL-1 receptor antagonist
Franzetti et al. (2020)	Italy	Case report	1; M	P/F: 50 mmHg	–	Anakinra		Day 7 after admission	100 mg/6 h	Subcutaneous	–	Lpv/Rtv, remdesivir	By day 16, a substantial improvement in the respiratory function of the patient was also noticed, with SaPO_2_ levels of 92% while on Venturi mask	–	This report highlights the high tolerability and the interesting immunomodulatory profile of anakinra in the setting of severe patients associated with remdesivir therapy
Cremer et al. (2021)	United States	MC, RCT, double-blind	40; 26 M:14 F	Baseline SOFA score, median (IQR): 2 (2–3); baseline P/F-mmHg: 137 (88–193)	CRP-mg/dl: 13.1 (9.8–18.8); ferritin-ng/ml: 1,040 (486–1860)	Mavrilimumab	Placebo	Time from symptom onset to hospitalization: 7 (4–8)	A single dose of 6 mg/kg	Intravenous	HTN (22), DB (17), HL (18), CAD (4)	Antiviral drugs, convalescent plasma, corticosteroids, other immunosuppressive agents	At 14 days, 12 patients in the mavrilimumab group were alive and off supplemental oxygen therapy compared with 9 patients in the placebo group	Adverse events were similar between groups. Treatment-related deaths were not observed	There was no significant difference in the proportion of patients alive and off oxygen therapy at day 14; despite the harm or benefit of mavrilimumab therapy in this patient population, it remains possible given the wide confidence intervals
De Luca et al. (2021)	Italy	SC, prospective cohort	39; 29 M:10 F	P/F-mmHg (KPa): mavrilimumab 196 (167–215), control 217 (138–258)	CRP-mg/L: mavrilimumab 152 (100–177), control 123 (77–190); ferritin, µg/L: mavrilimumab 2,302 (1,040–3,217), control 1,269 (854–3,369)	Mavrilimumab + standard of care	Standard of care	Fever duration (days): mavrilimumab 11 (10–12), control 7 (4–10)	A single dose of 6 mg/kg	Intravenous	–	HCQ, Lpv/Rtv, azithromycin	During the 28-day follow-up, 7 patients in the control group died, and no patient in the mavrilimumab group died. At day 28, 17 patients in the control group showed clinical improvement and all patients in the mavrilimumab group. Fever resolution was faster in mavrilimumab recipients *versus* controls	Mavrilimumab group with no infusion reactions; 3 patients in the control group developed infectious complications	Treatment of mavrilimumab was associated with improved clinical outcomes compared with standard care in non-MV patients with severe COVID-19 and systemic hyper-inflammation

MC, multi-center; SC, single-center; F, female; M, male; IL-6, interleukin-6; CRP, C-reactive protein; ALT, alanine aminotransferase; INR, international normalized ratio; PLT, platelet; AF, atrial fibrillation; CAD, coronary artery disease; COPD, chronic obstructive pulmonary disease; DB, diabetes; CKD, chronic kidney disease; HL, hyperlipidemia; CVI, cardiovascular impairment; CVD, cardiovascular disease; HTN, hypertension; HI, hepatic impairment; HF, heart failure; CA, cancer; CLD, chronic lung disease; CCD, chronic cardiac disease; CPD, chronic pulmonary disease; AMN, active malignant neoplasm; NIV, noninvasive ventilation; MV, mechanical ventilation; CI, confidence interval; TCB, tocilizumab; CP, cumulative percentage; SAE, serious adverse events; AE, adverse events; HCQ, hydroxychloroquine; Lpv/Rtv, lopinavir/ritonavir; IFN, interferon; HR, hazard ratio; OR, odds ratios; P/F, PaO_2_:FiO_2_; SOC, standard of care; KD, kidney disease; IPTW, inverse probability treatment weighting; DXMS, dexamethasone; P, patient; SOT/CTTR, solid organ and composite tissue transplant recipients; CCI, Charlson Comorbidity Index; suPAR, soluble urokinase plasminogen activator receptor.

### 3.4 Results of the Meta-analysis

We cannot conduct a quantitative analysis of anakinra, sarilumab, and mavrilimumab for some outcomes, owing to differences in outcomes reported, study design, and limited study numbers. Especially for mavrilimumab, only one RCT and one cohort met the inclusion criteria. If we could not perform a meta-analysis, we commented on the results from all included studies.

#### 3.4.1 Mortality Outcome (All-Cause Mortality at Days 28–30)

##### Tocilizumab

**FIGURE 2:**
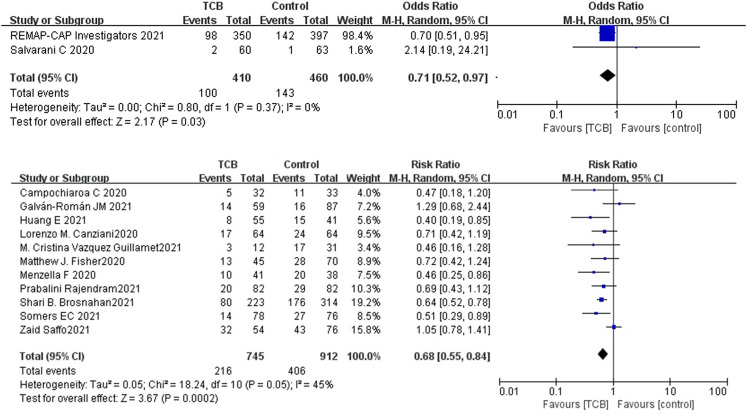
(**A**) Results from randomized controlled trials (RCTs): the mortality outcome of tocilizumab for COVID-19 (at the edge of sepsis).

Among the 14 controlled studies, one RCT and 6 cohorts neither reported a difference for mortality at days 28–30 between the tocilizumab and control groups. Compared to the control group, the results of RCTs showed that the use of tocilizumab for patients with COVID-19 (at the edge of sepsis) might decrease the mortality rate (OR 0.71, 95%CI: 0.52–0.97, *I* ^2^ = 0%), and there was a significant difference between the two groups (Figure 2A). The non-RCTs showed a similar result (RR 0.68, 95%CI: 0.55–0.84, *I* ^2^ = 45%), and there was statistical significance (Figure 2B).

##### Sarilumab

**FIGURE 3:**
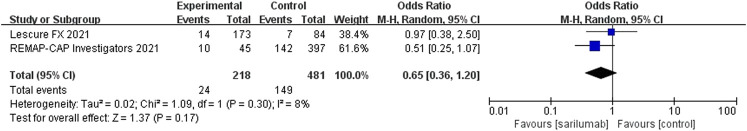
Results from randomized controlled trials: the mortality outcome of sarilumab for COVID-19 (at the edge of sepsis).

For sarilumab, of the studies that met the inclusion criteria, only two RCTs (one of the RCTs studied tocilizumab and sarilumab) and two non-RCTs provided data on mortality outcome. Among the two non-RCTs, one cohort did not set up a control group. Compared to the control group, the results of RCTs showed that the use of sarilumab for patients with COVID-19 (at the edge of sepsis) might reduce the mortality rate (OR 0.65, 95%CI: 0.36–1.2, *I* ^2^ = 8%), but there was no significant difference between the two groups (Figure 3). However, due to the lack of research, data synthesis for outcomes of non-RCTs was not conducted.

##### Anakinra

**FIGURE 4:**
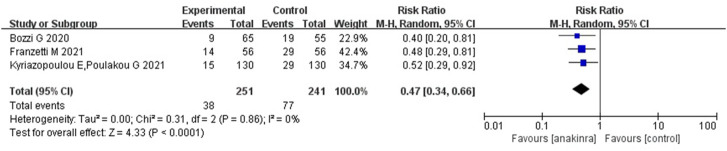
Results from non- randomized controlled trials: the mortality outcome of anakinra for COVID-19 (at the edge of sepsis).

For anakinra, of the studies that met the inclusion criteria, 1 RCT and 4 non-RCTs provided data on mortality outcome. Due to the insufficiency of RCTs, we only quantitatively synthesized the results of non-RCTs. Compared to the control group, the results of non-RCTs showed that the use of anakinra for patients with COVID-19 (at the edge of sepsis) might reduce the mortality rate (RR 0.47, 95%CI: 0.34–0.66, *I* ^2^ = 0%), and there was statistical significance (Figure 4).

##### Mavrilimumab

The only RCT, published in 2021, explored outcomes in 21 patients who received mavrilimumab and 19 patients who received placebo. The median (IQR) baseline SOFA score of enrolled patients was 2 (2 to 3). The study reported no significant association with the proportion of patients alive and off oxygen therapy at day 14. The other cohort, published in 2020, explored outcomes in 12 patients who received mavrilimumab and 26 patients who received standard of care. The median (IQR) P/F ratio of the mavrilimumab and control group was 196 (167–215) and 217 (138–258) mmHg, respectively. The study reported that mavrilimumab was associated with a reduced mortality rate and improved clinical outcomes. The benefits of mavrilimumab therapy for those patients remained uncertain, given the insufficient controlled studies and the small sample size.

#### 3.4.2 Safety Outcomes

Treatment-related adverse events (TRAEs) were reported in the majority of research and typically included neutropenia, secondary infections, increase in liver enzymes, and thromboembolism (Table 2). Due to the insufficient studies of safety outcome, we only conducted a quantitative synthesis for tocilizumab.

**TABLE 2:** Adverse events (AEs) summarized from controlled studies.

Author	Immunomodulator	AEs (percentages)
Salvarani et al. (2021)	Tocilizumab	Control group: 2 severe infections; treatment group: 1 upper gastrointestinal tract bleeding. The most common adverse events were increased alanine aminotransferase level and decreased neutrophil count
REMAP-CAP Investigators et al. (2021)	Tocilizumab, sarilumab	Treatment group: 1 secondary bacterial infection, 5 bleeding events, 2 cardiac events, 1 deterioration in vision. Control group: 4 bleeding events, 7 thromboses
Canziani et al. (2020)	Tocilizumab	Thrombosis: treatment group (19%), control group (17%). Bleeding: treatment group (17%), control group (13%). Infection: treatment group (31%), control group (39)
Fisher et al. (2021)	Tocilizumab	No observed increased risk of secondary infection within 14 days of treatment with tocilizumab
Campochiaroa *et al*. (2020)	Tocilizumab	Pulmonary thrombosis: treatment group (6%), control group (9%). Raised ALT, AST level: treatment group (15%), control group (18%). Neutropenia: treatment group (16%), control group (0)
Vazquez Guillamet et al. (2021)	Tocilizumab	Culture-negative sepsis: treatment group (41.7%), control group (19.4)
Rajendram et al. (2021)	Tocilizumab	Secondary infection: treatment group (25.6%), control group (25.6%)
Huang et al. (2021)	Tocilizumab	Secondary infection: treatment group (31%), control group (17%)
Saffo et al. (2021)	Tocilizumab	Bleeding: treatment group (24.1%), control group (14.5%). Blood stream infection: treatment group (7.4%), control group (9.2%). Pulmonary infection (endotracheal aspirates/sputum): treatment group (25.9%), control group (30.3%)
Somers et al. (2021)	Tocilizumab	Superinfection: treatment group (54%), control group (26%). Bloodstream infection: treatment group (14%), control group (9%). Pneumonia: treatment group (45%), control group (20%)
Brosnahan et al. (2021)	Tocilizumab	Positive blood culture: combination group (steroid + tocilizumab) (11.6%), steroid group (12.7%). Positive Fungitell test: combination group (6.9%), steroid group (10.4%). Positive T2Candida panels: combination group (6.4%), steroids group (6.9%). Cytomegalovirus viral loads elevated: combination group (3.5%), steroids group 4.6%
Lescure et al. (2021)	Sarilumab	Serious infection: treatment group (12%), control group (12%). ALT increase: treatment group (31.02%), control group (19%). Invasive bacterial or fungal infection: treatment group (6.9%), control group (4%). Grade ≥2 hypersensitivity reaction: treatment group (2.4%), control group (0%). Grade 4 neutropenia: treatment group (2.7%), control group (0)
Della-Torre et al. (2020)	Sarilumab	Infections: treatment group (21%), control group (18%). Neutropenia: treatment group (14%), control group (0). Increase in liver enzymes: treatment group (14%), control group (0). Thromboembolism: treatment group (7%), control group (7%)
Kyriazopoulou et al. (2021a)	Anakinra	Infections and infestations: treatment group (8.4%), control group (15.9%). Anemia: treatment group (14.3%), control group (19.6%). Increase of liver function tests: treatment group (35.8%), control group (33.3%). Hyperglycemia: treatment group (36.5%), control group (40.2%). Hyponatremia: treatment group (7.9%), control group (12.2%). Hypernatremia: treatment group (11.4%), control group (9%)
Bozzi et al. (2021)	Anakinra	Treatment group: grade ≥3 GGT increase (27.7%), anemia (24.6%), ALT increase (6.2%), granulocytopenia (1.5%). Control group: a comparable proportion of these AEs
Franzetti et al. (2021)	Anakinra	Bloodstream infections: treatment group (16%), control group (7.1%). Urinary tract infections: treatment group (3.5%), control group (1.8%). Pneumonia infections: treatment group (7.1%), control group (7.1%)
Kyriazopoulou et al. (2021b)	Anakinra	Electrolyte abnormalities: treatment group (26.9%), control group (31.5%). Elevated liver function tests: treatment group (30.8%), control group (39.2%). Gastrointestinal disturbances: treatment group (11.5%), control group (6.9%). Anemia: treatment group (16.9%), control group (20%)
Cremer et al. (2021)	Mavrilimumab	Bacterial pneumonia: treatment group (10%), control group (5%). SAEs: treatment group (24%), control group (21%). Circulatory shock: treatment group (10%); control group (5%); Acute kidney injury: treatment group (19%), control group (16%). ALT ≥3ULN: treatment group (24%), control group (16%). AST ≥3ULN: treatment group (29%), control group (21%)
De Luca et al. (2020)	Mavrilimumab	Infectious complications: treatment group (0), control group (12%)

ALT, alanine aminotransferase; AST, aspartate aminotransferase; GGT, gamma-glutamyl transferase; ULN, upper limit of normal.

##### Tocilizumab

**FIGURE 5:**
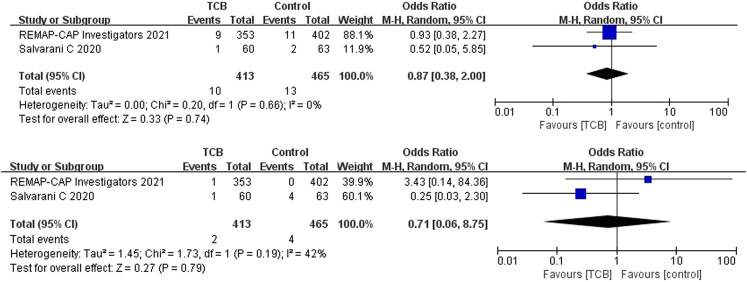
(**A**) Results from randomized controlled trials (RCTs): the serious adverse events (SAEs) of tocilizumab for COVID-19 (at the edge of sepsis).

**FIGURE 6:**
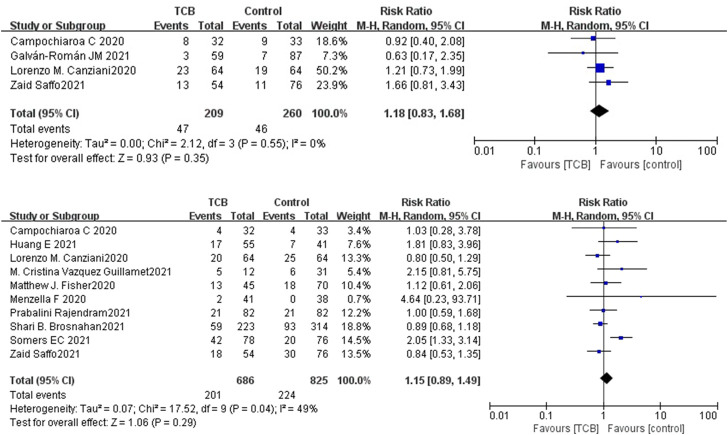
(**A**) Results from non-randomized controlled trials (non-RCTs): the serious adverse events of tocilizumab for COVID-19 (at the edge of sepsis).

Both 2 RCTs reported SAEs and secondary infections; 4 of 11 non-RCTs reported SAEs and 10 reported secondary infections. Tocilizumab was associated with less SAEs (OR 0.87, 95%CI: 0.38–2.00, *I* ^2^ = 0%) and lower rates of secondary infections (OR 0.71, 95%CI: 0.06–8.75, *I* ^2^ = 42%) compared with the control groups, which both did not reach significance in RCTs (Figures 5A,B). For non-RCTs, tocilizumab was associated with slightly more SAEs (RR 1.18, 95%CI: 0.83–1.68, *I* ^2^ = 0) and secondary infections (RR 1.15, 95%CI: 0.89–1.49, *I* ^2^ = 49%) compared with the control arm, but there was no statistical significance (Figures 6A,B).

##### Other Anti-cytokine Agents

The included RCTs reported that the incidence of treatment-emergent SAEs through day 28 was higher in the placebo and standard-of-care group (21.2%) compared to the anakinra and standard-of-care group (16.5%). The non-serious TRAEs were similar in both treatment groups (Kyriazopoulou et al., 2021a). Only two cohorts reported secondary infection outcomes, and none reported SAEs. Both Franzetti M *et al*. and Bozzi G *et al*. reported that the rate of adverse events related to infection (or bloodstream infections) was similar between groups—for example, 26.8% occurred in the anakinra group and 16.1% in the control group (Bozzi et al., 2021; Franzetti et al., 2021). Among these infectious events, 9/56 developed bloodstream infections in the anakinra group and 4/56 in the control group (Franzetti et al., 2021). Meanwhile, they all suggested that special attention should be paid to possible infective reactivations or bacterial sepsis due to anakinra. In studies with a comparator arm exploring outcomes from patients who received mavrilimumab or sarilumab, the frequency of TRAEs was similar in both treatment and comparator groups.

### 3.5 Quality of Evidence

For mortality outcomes, the quality of evidence of tocilizumab for COVID-19 (at the edge of sepsis) was of low and very low quality for RCTs and non-RCTs, respectively. Meanwhile, the quality of evidence of sarilumab and anakinra for COVID-19 (at the edge of sepsis) was of low and very low quality, respectively. As for the SAEs and secondary infections of tocilizumab for COVID-19 (at the edge of sepsis), the quality of evidence was all low for RCTs and very low for non-RCTs, respectively. The results are shown in Supplementary Table S8 (Supplementary Material S2, Appendix p43–45).

## 4 Discussion

In terms of etiology, sepsis can be classified as bacterial sepsis, fungal sepsis, and viral sepsis based on different pathogens. Sepsis patients with a SOFA score of 2 or more in a general hospital population with presumed infection had an increased risk of death by 2–25 times compared to patients with a SOFA score of less than 2 (Singer et al., 2016; Seymour et al., 2016). The population included in this study was COVID-19 patients with SOFA score ≥2, who were already in the state of sepsis or were about to deteriorate into sepsis, and these patients urgently needed appropriate, safe, and effective treatment. In this study, we evaluated the efficacy and safety of tocilizumab, sarilumab, siltuximab, anakinra, mavrilimumab, and lenzilumab to provide relevant clinical evidence and research ideas for treatment.

### 4.1 Anti-cytokine Therapy

The local inflammatory response caused by an infection can promote the replacement of damaged tissues by new tissues and play a role in weakening the damage that has occurred, but when excessive inflammation occurs, it may cause systemic inflammatory response syndrome (SIRS) and lead to sepsis. Therefore, timely detection of cytokine storms and proper regulation of inflammatory response may be of great significance to the prevention of sepsis. The “Expert Consensus on Early Prevention and Blocking of Sepsis in China” recommended that when infected patients experience significant increases in cytokines or inflammatory imbalances, the inflammation should be adjusted as soon as possible using glucocorticoids, nonsteroidal anti-inflammatory drugs, traditional Chinese medicine preparations, antibodies targeting inflammatory mediators, *etc*. (Emergency medicine branch of CPAM et al., 2020). Many studies showed that the factors mainly involved in SIRS and compensatory anti-inflammatory response syndrome include TNF-α, IL-1, IL-6, *etc*. The Expert Consensus suggested that, for patients with high-risk sepsis infection, cytokine monitoring should be carried out regularly (2–4-h repetition) to find suspected sepsis patients in time. At present, the cytokine commonly detected in hospitals is IL-6. As a cytokine, IL-6 mainly stimulates the proliferation and differentiation of cells involved in immune response and plays an important role in the anti-infection immune response (Emergency Medicine Branch of CPAM et al., 2020).

IL-6 inhibitors include tocilizumab, sarilumab and siltuximab. Tocilizumab and sarilumab were approved for rheumatoid arthritis, and siltuximab was approved for Castleman’s disease. The IL-1 receptor antagonist (anakinra) is a cornerstone treatment for hyperinflammatory conditions such as Still’s disease. Some studies showed that cytokine-directed agents such as IL-6 and IL-1 inhibitors might be effective in the treatment of cytokine storm syndromes, including macrophage activation syndrome and cytokine release syndrome (La Rosée et al., 2019). The GM-CSF blockade included mavrilimumab and lenzilumab, which is designed to prevent and treat cytokine storm (De Luca et al., 2020; Aroldi et al., 2019). This systematic review identified and summarized RCTs, non-RCTs, and case reports (series) to evaluate the effect and safety of tocilizumab, sarilumab, siltuximab, anakinra, mavrilimumab, and lenzilumab. The meta-analysis results showed that tocilizumab might reduce the mortality of patients with COVID-19 (at the edge of sepsis) (RCTs: OR: 0.71, 95%CI: 0.52–0.97, low-certainty evidence; non-RCTs: RR: 0.68, 95%CI: 0.55–0.84, very low-certainty evidence) as was anakinra (non-RCTs: RR: 0.47, 95%CI: 0.34–0.66, very low-certainty evidence). Sarilumab might reduce the mortality of patients with COVID-19 (at the edge of sepsis), but there was no statistical significance (OR: 0.65, 95%CI: 0.36–1.2, low-certainty evidence). For safety outcomes, whether tocilizumab had an impact on SAEs was very uncertain (RCTs: OR: 0.87, 95%CI: 0.38–2.0, low-certainty evidence; non-RCTs: OR: 1.18, 95%CI: 0.83–1.68, very low-certainty evidence) as was on secondary infections (RCTs: OR: 0.71, 95%CI: 0.06–8.75, low-certainty evidence; non-RCTs: RR: 1.15, 95%CI: 0.89–1.49, very low-certainty evidence).

### 4.2 Special Population

At present, there are still few large-scale randomized controlled prospective studies on COVID-19 (at the edge of sepsis). The experiences of case or case series still have a certain reference significance for clinical treatment, especially for the individualized treatment of special populations, such as critically ill children, immunocompromised individuals, and elderly patients with a variety of chronic diseases. Patel PA *et al*. reported a case of severe pediatric COVID-19 presenting with respiratory failure and severe thrombocytopenia. On day 7, because of continued fever and elevated inflammatory markers, remdesivir and tocilizumab were given. On the next day, she had significant clinical improvement, so the treatment with cytokine-directed agents may be considered in critically ill patients (Patel et al., 2020).

Patients with impaired immune function are more at risk in case of adverse outcomes. Leelayuwatanaku N *et al*. reported two patients (P/F < 300 mmHg) with human immunodeficiency virus (HIV) infection and multiple myeloma relapse, respectively. After tocilizumab, hemoperfusion, and immunoglobulin comprehensive treatment, their P/F levels increased significantly, and they survived to discharge (Leelayuwatanaku et al., 2021). In addition, Kataoka H *et al*. reported an 85-year-old patient with Sjögren’s syndrome, whose P/F decreased to 100 mmHg. After receiving a single dose of tocilizumab, the symptoms improved. This patient represents a supplementary case confirming the safety and efficacy of tocilizumab for elderly COVID-19 patients with autoimmune diseases. It is also suggested that combination therapy may be a promising treatment for severe COVID-19 in immunocompromised hosts (Kataoka et al., 2021).

The experience of COVID-19 patients with solid organ and composite tissue transplantation has not been reported in detail before. Morillas JA *et al*. reported 5 patients with COVID-19 (P/F < 300 mmHg) who received kidney transplantation, lung transplantation, face transplantation, and liver transplantation, respectively. These patients also had chronic diseases, such as heart diseases, bladder cancer, rheumatic heart disease, *etc*. Their C-reactive protein (CRP) levels decreased significantly within a few days after the application of tocilizumab. The findings showed that tocilizumab could be used without major direct toxicity in solid organ and composite tissue transfer recipients early after initiation of mechanical exploitation due to COVID-19, regardless of the type of organ transferred. However, the authors suggested that the diagnosis and side effects need to be further studied (Morillas et al., 2020). Ladna M *et al*. and Thammathiwa T *et al*. shared the treatment experiences of transplant patients, respectively. One patient who received a kidney and heart transplant in February 2020 had a relatively poor clinical condition with a P/F level of 117 mmHg. On day1, he was given a dose of 400 mg of tocilizumab, broad-spectrum antibiotics, and hydroxychloroquine, and transplant immunosuppression with tacrolimus was continued. After 11 days of treatment, he was discharged without supplemental oxygen requirement (Ladna et al., 2021; Thammathiwat et al., 2021).

In addition to tocilizumab treatment cases, there are few case reports on IL-1 receptor antagonist. Filocamo G *et al*. and Franzetti M *et al*. in Italy treated two severe patients (P/F <200 mmHg) with IL-1 receptor antagonist anakinra. These studies suggested that, in the cytokine storm occurring during severe COVID-19 pneumonia, the high tolerability, short half-life, and immunomodulatory profile of anakinra may be useful. IL-1 inhibition may represent a safe and promising strategy to reduce inflammation, thus preventing multi-organ dysfunction (Filocamo et al., 2020; Franzetti et al., 2020).

### 4.3 Limitation

First, the lack of RCTs limited our analyses. Some included studies were case reports or series and had no proper control groups. Meanwhile, some articles of which the full texts or data were not accessible and those in languages other than Chinese and English were excluded from the analysis. This might have led to overlooking some critical findings or observations. In addition, in this study, the SOFA score or related indicators of some patients included in the study were median or mean, so not all patients were septic patients, but the results of this population also reflected a trend problem because some patients might be or would be in a state of sepsis. Thirdly, we found that most patients use antiviral drugs, glucocorticoids, immunoglobulins, plasma, broad-spectrum antibiotics, and other drugs at the same time. We cannot rule out the impact of these drugs on the disease**.**

## 5 Conclusion

The results of this systematic review showed that tocilizumab, sarilumab, and anakinra might reduce the mortality of people with COVID-19 (at the edge of sepsis), and tocilizumab did not significantly affect SAEs and secondary infections. However, given the limited clinical researches and low-quality evidence, this conclusion needs more clinical evidence to be verified. In addition, so far, there is still no unified opinion on the timing, dosage, usage, and applicable population of these drugs all over the world, which also adds to the uncertainty of the conclusion of this study.

## Data Availability

The original contributions presented in the study are included in the article/Supplementary Material. Further inquiries can be directed to the corresponding author.
