# Supplementary material for: Specific Interleukin-1 Inhibitors, Specific Interleukin-6 Inhibitors, and GM-CSF Blockades for COVID-19 (at the Edge of Sepsis): A Systematic Review
Source: Front Pharmacol. 2022 Jan 21;12:804250. doi: 10.3389/fphar.2021.804250 (PMC8815770; doi:10.3389/fphar.2021.804250)
Supplement: Supplementary file 1 [file DataSheet1.doc]

| **Section / topic** | **#** | **Checklist item** | **Reported on page #** |
| --- | --- | --- | --- |
| **TITLE** | | |  |
| Title | 1 | Specific interleukin-1 inhibitors, specific interleukin-6 inhibitors and GM-CSF blockades for COVID-19 (at the edge of sepsis): a systematic review | Title |
| **ABSTRACT** | | |  |
| Structured summary | 2 | During the COVID-19 pandemic, some severe and critically ill COVID-19 patients with multiple organ dysfunction could developed characteristics typical of sepsis and met the diagnostic criteria for sepsis. Timely detection of cytokine storm and appropriate regulation of inflammatory response may be significant in the prevention and treatment of sepsis. This study evaluated the efficacy and safety of specific interleukin-1 inhibitors, specific interleukin-6 inhibitors and GM-CSF blockade in the treatment of COVID-19(at the edge of sepsis) patients through systematic review and meta-analysis. | Abstract |
| **INTRODUCTION** | | |  |
| Rationale | 3 | Sepsis is a dangerous disease with high mortality and high treatment cost. According to an article published in The Lancet in 2020, the number of sepsis patients worldwide reached 48.9 million in 2017, among which 11 million patients died, accounting for one-fifth of the global death toll.  During the COVID-19 pandemic, patients with severe or critically ill COVID-19 may develop circulation disorders and severe lung damage. Some patients with multiple organ dysfunction, such as liver and kidney, show typical characteristics of sepsis and meet the diagnostic criteria for sepsis. According to Sepsis-3, sepsis was defined as life-threatening organ dysfunction caused by a dysregulated host response to infection. The organ dysfunction can be represented by an increase in the Sequential [Sepsis-related] Organ Failure Assessment (SOFA) score of 2 points or more, which is associated with an in-hospital mortality greater than 10%). Recent studies have shown that patients with severe and critical diseases may experience immune hyperactivity with increased levels of including interleukin (IL)-1, IL-6, granulocyte-monocyte stimulating factor (GM-CSF), interferon-γ-inducible protein 10 (IP-10), tumor necrosis factor-α (TNF-α) and other several inflammatory cytokines, and are associated with adverse clinical outcomes. Therefore, inhibition of proinflammatory cytokines may be a potential therapeutic strategy in COVID-19 patients with sepsis. | Introduction |
| Objectives | 4 | This study is the first to screen COVID-19 patients with sepsis or at the edge of sepsis through SOFA score, and the efficacy and safety of anti-cytokine therapy, such as specific IL-1, IL-6 inhibitors and anti-GM-CSF in COVID-19 patients with organ dysfunction (SOFA≥2) were systematically reviewed.  Intervention(s)：Specific interleukin-1 inhibitors, specific interleukin-6 inhibitors and GM-CSF blockade  Comparator(s)/control: Placebo, or gave patients standard treatment without Specific interleukin-1 inhibitors, specific interleukin-6 inhibitors or GM-CSF blockade.  Outcome(s): mortality and safety outcomes include serious adverse events and secondary infection.  Study: Randomized clinical trials, cohort studies, case-control studies, case series, case reports, clinical guidelines, protocols for clinical trials, any other grey literature will be included. | Introduction |
| **METHODS** | | |  |
| Protocol and registration | 5 | The study protocol was registered with the National Institute for Health Research international prospective register of systematic reviews.  Citation: Ying Wang, Rulin Dai, Xin Lv, Qian Yu, Kun Zhu. Specific interleukin-1 inhibitors, specific interleukin-6 inhibitors and anti-GM-CSF for COVID-19 (at the edge of sepsis): a living system review. PROSPERO 2020 CRD42020226545 Available from: https://www.crd.york.ac.uk/prospero/display_record.php?ID=CRD42020226545 | Materials and Methods |
| Eligibility criteria | 6 | (1) patients were confirmed SARS-CoV-2 infection and their Sequential Organ Failure Assessment score(SOFA, including mean value, median, and absolute value) ≥2, or in the SFOA scoring tool, a certain system index (including mean, median, and absolute value) should be within the range corresponding to the system score≥2, A certain system indicator (including mean, median, absolute value, and absolute value) shall be within the range corresponding to the system score≥2, for example, PaO2/FiO2 (absolute value, mean value, or median value) should be less than 300mmHg. SpO_2_/FiO_2_ ratio of 315 corresponded with a PaO_2_/FiO_2_ ratio of 300mmHg[ SpO_2_/FiO_2_=64+0.84*(PaO_2_/FiO_2_)] . In this review, we defined such COVID-19 patients were at the edge of sepsis. (2) The intervention of interest was specific interleukin-1 inhibitors(anakinra), specific interleukin-6 inhibitors (tocilizumab, siltuximab, sarilumab) and anti-GM-CSF(mavrilimumab, lenzilumab) with or without standard of care (or treatment), glucocorticoids. Comparator treatments included placebo, standard of care (or treatment), glucocorticoids, or no intervention; studies with no comparator group were also included. (3) Randomized clinical trials, quasi-experimental studies, cohort studies, case series, case reports, clinical guidelines, protocols for clinical trials, any other grey literature will be included. Studies will not be limited in terms of country. | Materials and Methods |
| Information sources | 7 | Electronic searches were carried out in PubMed, EMBASE,Cochrane Library, Clinical Key, Wanfang Database; China National Knowledge Infrastructure (CNKI). The search terms we used were “SARS-CoV-2”, “Corona Virus Disease 2019”, “COVID-19”, “anakinra”, “tocilizumab”, “siltuximab”, “sarilumab”, “mavrilimumab”, “lenzilumab” and relevant key words for publications published until 22.8.2021.  Other available resources were also used to identify relevant articles. The language will be limited to Chinese and English. Eligible articles were identified for inclusion by screening the titles, abstracts, and full text. Other relevant studies were manually screened by investigators from the reference list of included studies for further analysis. There is no date limit. | Materials and Methods |
| Search | 8 | The search strategy is attached. | Materials and Methods |
| Study selection | 9 | State the process for selecting studies (i.e., screening, eligibility, included in systematic review, and, if applicable, included in the meta-analysis).  Following the removal of duplicate entries, a three-stage screening process was followed to identify eligible records through the sequential examination of each title, abstract, and full text. Two reviewers(Y.W and K.Z) screened each record, with provision for arbitration from a third reviewer(Q.Y). | Materials and Methods |
| Data collection process | 10 | Describe method of data extraction from reports (e.g., piloted forms, independently, in duplicate) and any processes for obtaining and confirming data from investigators.  Two independent reviewers(Y.W and K.Z) carried out search in a standardized process, followed to identify eligible records through the sequential examination of each title, abstract, and full text. Disagreements were resolved by consensus and unresolved conflicts were decided by a third reviewer(Q.Y). | Materials and Methods |
| Data items | 11 | List and define all variables for which data were sought (e.g., PICOS, funding sources) and any assumptions and simplifications made.  Data will be extracted from the eligible studies using a template by two independent reviewers (Y.W and K.Z) and validated by a third (Q.Y). The following information will be extracted: Published year, authors and country of the study, study design, sample size, participant demographics, time of administration, intervention characteristics (name of agent, dose, route), concomitant medications, survival outcome, treatment related adverse events, and authors’ conclusions. | Materials and Methods |
| Risk of bias in individual studies | 12 | Describe methods used for assessing risk of bias of individual studies (including specification of whether this was done at the study or outcome level), and how this information is to be used in any data synthesis.  Included studies were assessed the potential bias by two reviewers (RL.D and R.L)independently. The third researcher (X.L) was consulted for resolving any difference of opinion. The National Institute for Health and Care Excellence’s Quality Assessment for Case Series will be used to evaluate the quality of case series. The total score is 8 points, in which a score of 4–8 is high quality, and a score less than 4 is low quality.  The methodological quality for cohort and case-control studies were assessed based on the Newcastle-Ottawa Scale(NOS) .The methodological quality of randomized clinical trials (RCTs) were assessed based on the ‘Risk of Bias’ 2.0 tool . Each checklist item was judged as ‘low’, ‘moderate’, ‘serious’ and ‘critical’. The quality of evidence was assessed by using the ‘Grading of Recommendations Assessment, Development and Evaluation (GRADE)’ tool. The quality of evidence of each outcome is classified as ‘high’, ‘moderate’, ‘low’ or ‘very low’. | Materials and Methods |
| Summary measures | 13 | State the principal summary measures (e.g., risk ratio, difference in means).  For dichotomous outcomes, the number of events and total number of participants in two groups were recorded. Fixed-effects model was used if the result of the Q test was significant (p＞0.1) or I2＜50%. But the different types of studies were analyzed separately (such as RCTs and controlled NRSIs). If we could not perform a meta-analysis, we had planned to comment on the results as a narrative with the results from all studies presented in tables. The odd’s ratio(OR) and the risk ratio(RR) with 95% confidence intervals(CIs) was assessed for RCTs and controlled NRSIs respectively. | Materials and Methods |
| Synthesis of results | 14 | Describe the methods of handling data and combining results of studies, if done, including measures of consistency (e.g., I^2^) for each meta-analysis.  A Chi^2^ test with a significance level at P≤0.1 was used to assess heterogeneity of treatment effects between trials. The I^2^ statistic was used to quantify possible heterogeneity (I^2^ statistic: 30% to 60% may represent moderate heterogeneity, 75% to 100% considerable heterogeneity). If heterogeneity had been above 80%, we would explore potential causes through sensitivity and subgroup analyses. If we had not found a reason for heterogeneity, we would not have performed a meta-analysis. | Materials and Methods |

Page 1 of 2

| **Section/topic** | **#** | **Checklist item** | **Reported on page #** |
| --- | --- | --- | --- |
| Risk of bias across studies | 15 | Specify any assessment of risk of bias that may affect the cumulative evidence (e.g., publication bias, selective reporting within studies).  The methodological quality for cohort and case-control studies were assessed based on the Newcastle-Ottawa Scale. The total score is 9 points, in which a score of 0-3, 4-6, 7-9 were, respectively, considered as low, moderate, and high quality.  The National Institute for Health and Care Excellence’s Quality Assessment for Case Series will be used to evaluate the quality of case series. The total score is 8 points, in which a score of 4-8 is high quality, and a score less than 4 is low quality. | Materials and Methods |
| Additional analyses | 16 | Describe methods of additional analyses (e.g., sensitivity or subgroup analyses, meta-regression), if done, indicating which were pre-specified.  Subgroup analyses will be conducted, if appropriate based on the data retrieved. | Materials and Methods |
| **RESULTS** | | |  |
| Study selection | 17 | Give numbers of studies screened, assessed for eligibility, and included in the review, with reasons for exclusions at each stage, ideally with a flow diagram.  The included 43 studies were identified and critically evaluated, which included a total of 5003 patients with confirmed SARS-CoV-2 infection, of whom 3289 received mechanical ventilation. | Results |
| Study characteristics | 18 | For each study, present characteristics for which data were extracted (e.g., study size, PICOS, follow-up period) and provide the citations.  Only 11 studies reported SOFA scores (median or mean) of enrolled patients, of which 4 studies reported SOFA scores greater than or equal to 6 (tocilizumab), 3 studies reported scores between 4 and 5 (tocilizumab) and 4 studies reported scores between 2 and 3 (3 for Anakinra, 1 for Mavrilimumab, and the rest for Tocilizumab). The remaining 32 articles reported patients' respiratory status, including PaO2/FiO2 ratio(P/F) or SpO2/FiO2 ratio(median or mean value) and platelet, of which 5 studies included patients with P/F ratios less than or equal to 100mmHg，and of which 13 studies reported patients with P/F ratio between 100-200mmHg.  Most patients received standard care (or standard care) based on local treatment guidelines. However, the medication regimens of standard of care were different, mainly including antiviral drugs, antibiotics, glucocorticoids and other symptomatic drugs. Immunomodulatory drugs were mainly used by intravenous injection, and a few studies were in the form of subcutaneous injection. In addition, there is still no consensus on the dosage of immunomodulatory drugs for such patients until now. In the included articles, the dosage of most patients were: tocilizumab 8mg/kg/dose up to a maximum of 800 mg, sarilumab 400mg/dose with 1-2 doses, anakinra 100mg/dose with 1-4 times a day, and mavrilimumab 6mg/kg/dose. Characteristics of included studies are presented in table 1. | Results |
| Risk of bias within studies | 19 | Present data on risk of bias of each study and, if available, any outcome level assessment (see item 12).  The methodological of RCTs were low to moderate respectively, the results were showed in figure S1(suppletary2,appendix p1)  Some studies reported only one outcome, and we assess the risk of bias for the results reported in the study. For example, bias in measurement of outcomes was not applicable for safety for Shari B. Brosnahan, because they did not report this outcome. For mortality outcomes, the methodological quality of 16 cohorts were moderate to high, and 2 case-control studies were moderate. For safety outcomes, the methodological quality of 14 cohorts were low to high, and 2 case-control studies were low to moderate(NOS assessments results were showed in supplementary 2, appendix p2-41). The methodological quality evaluation results of the included case reports(series) showed that the quality was low to moderate (the results of quality were showed in supplementary 2, appendix, p42-43). | Results |
| Results of individual studies | 20 | For all outcomes considered (benefits or harms), present, for each study: (a) simple summary data for each intervention group (b) effect estimates and confidence intervals, ideally with a forest plot.  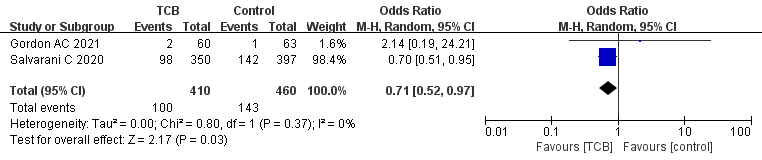  Figure 2a Results from RCTs: the mortality outcome of tocilizumab for COVID-19(at the edge of sepsis)  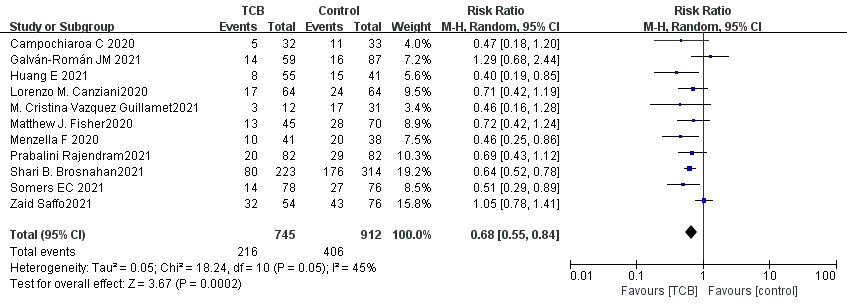  Figure 2b Results from cohorts and case-control studies: the mortality outcome of tocilizumab for COVID-19(at the edge of sepsis)  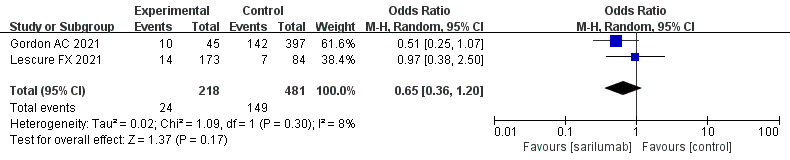  Figure 3 Results from RCTs: the mortality outcome of Sarilumab for COVID-19(at the edge of sepsis)  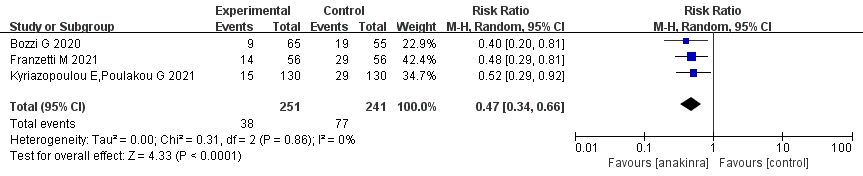  Figure 4 Results from cohorts: the mortality outcome of anakinra for COVID-19(at the edge of sepsis)  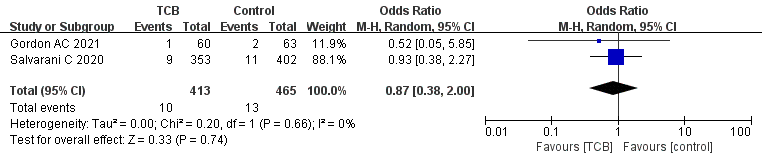  Figure 5a Results from RCTs: the SAEs of tocilizumab for COVID-19(at the edge of sepsis)  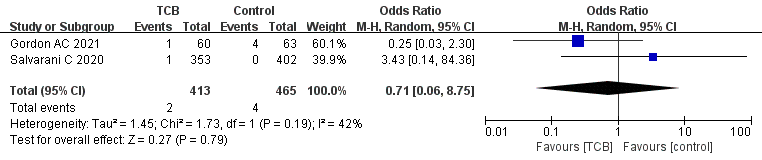  Figure 5b Results from RCTs: the secondary infections of tocilizumab for COVID-19(at the edge of sepsis)  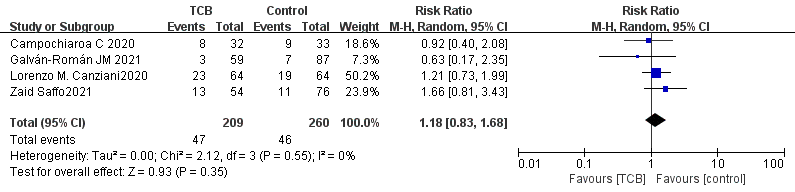  Figure 6a Results from cohorts and case-control studies: the SAEs of tocilizumab for COVID-19(at the edge of sepsis)  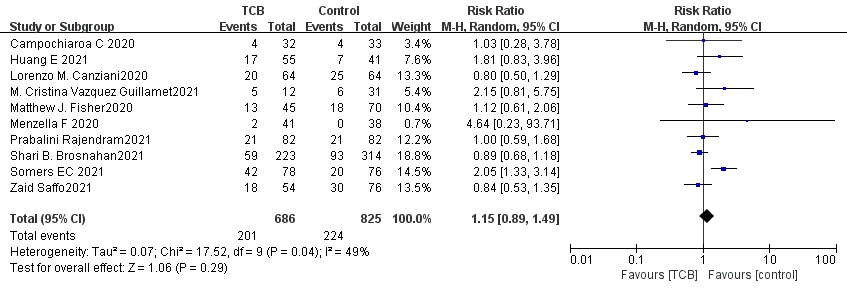  Figure 6b Results from cohorts and case-control studies: the secondary infections of tocilizumab for COVID-19(at the edge of sepsis) | Results |
| Synthesis of results | 21 | Present results of each meta-analysis done, including confidence intervals and measures of consistency.  This systematic review identified and summarized RCTs, non-RCTs(cohorts, case-control studies and case reports(series)) to evaluate the effect and safety of tocilizumab, sarilumab, siltuximab, anakinra, mavrilimumab and lenzilumab. Meta-analysis results showed that, tocilizumab may reduce may reduce the mortality of patients with COVID-19(at the edge of sepsis) (RCTs: OR 0.71, 95%CI 0.52-0.97, low-certainty evidence; non-RCTs: RR 0.68, 95%CI 0.55-0.84, very low-certainty evidence), as is anakinra(non-RCTs: RR 0.47,95%CI 0.34-0.66, very low-certainty evidence). Sarilumab may reduce the mortality of patients with COVID-19(at the edge of sepsis),but there was no statistically significant(OR 0.65,95%CI 0.36-1.2, low-certainty evidence).For safety outcomes, whether tocilizumab has an impact on SAEs is very uncertain (RCTs: OR 0.87,95%CI 0.38-2.0, low-certainty evidence; non-RCTs 1.18, 95%CI 0.83-1.68, very low-certainty evidence), as is on secondary infections(RCTs: OR 0.71,95%CI 0.06-8.75, low-certainty evidence; non-RCTs: RR 1.15, 95% CI 0.89-1.49, very low-certainty evidence). | Results |
| Risk of bias across studies | 22 | Present results of any assessment of risk of bias across studies (see Item 15).  We assessed the methodological quality and risk of bias for RCTs, cohorts,case-control studies and case reports(series) for different outcomes (such as mortality and safety) respectively, The methodological of RCTs were low to moderate respectively, the results were showed in figure S1(suppletary2,appendix p1)  Some studies reported only one outcome, and we assess the risk of bias for the results reported in the study. For example, bias in measurement of outcomes was not applicable for safety for Shari B. Brosnahan, because they did not report this outcome. For mortality outcomes, the methodological quality of 16 cohorts were moderate to high, and 2 case-control studies were moderate. For safety outcomes, the methodological quality of 14 cohorts were low to high, and 2 case-control studies were low to moderate(NOS assessments results were showed in supplementary 2, appendix p2-41). The methodological quality evaluation results of the included case reports(series) showed that the quality was low to moderate (the results of quality were showed in supplementary 2, appendix, p42-43). | Results |
| Additional analysis | 23 | Give results of additional analyses, if done (e.g., sensitivity or subgroup analyses, meta-regression [see Item 16]).  None | Results |
|  |  |  |
| **DISCUSSION** | | |  |
| Summary of evidence | 24 | Summarize the main findings including the strength of evidence for each main outcome; consider their relevance to key groups (e.g., healthcare providers, users, and policy makers).  Sepsis is divided into bacterial sepsis, fungal sepsis and viral sepsis based on different pathogens.According to sepsis-3.0, sepsis is infection with SOFA score≥2, the overall mortality risk is approximately 10% in patients with a SOFA score of 2 or more in a general hospital population with presumed infection, and an increased risk of death by 2–25 times compared to patients with a SOFA score of less than 2. The population included in this study was COVID-19 patients with SOFA score≥2, who were already in the state of sepsis or were about to deteriorate into sepsis, and these patients urgently needed appropriate, safe and effective treatment. In this study, we evaluated the efficacy and safety of tocilizumab, sarilumab, siltuximab, anakinra, mavrilimumab and lenzilumab to provide clinical evidence and research ideas for treatment.  The local inflammatory response caused by infection can promote the replacement of damaged tissue by new tissue and play a role in weakening the damage that has occurred. But when excessive inflammation occurs, it may cause systemic inflammatory response syndrome (SIRS) and lead to sepsis. Therefore, timely detection of cytokine storms and proper regulation of inflammatory response are of great significance to the prevention of sepsis. "Expert consensus on early prevention and blocking of sepsis in China" suggests that when infected patients find that cytokines are significantly elevated or inflammation is unbalanced, inflammation should be regulated as soon as possible. Including glucocorticoids, non hormonal anti-inflammatory drugs, traditional Chinese medicine preparations, inflammatory mediator specific antibodies, etc. Many studies believe that the factors mainly involved in SIRS and compensatory anti-inflammatory response syndrome (cars) include TNF- α, IL-1, IL-6, etc. The expert consensus suggested that for patients with high-risk sepsis infection, cytokine monitoring should be carried out regularly (2 ~ 4H repetition) to find suspected sepsis patients in time. At present, the cytokine commonly detected in hospitals is IL-6. As a cytokine of IL, IL-6 mainly stimulates the proliferation and differentiation of cells involved in immune response and plays an important role in the body's anti infection immune response.  Interleukin-6 inhibitors include tocilizumab, sarilumab and siltuximab, tocilizumab and sarilumab were approved for Rheumatoid Arthritis, and sarilumab was approved for Castleman’s disease. The interleukin-1 receptor antagonist (anakinra) is a cornerstone treatment for hyperinflammatory conditions such as Still's disease. Some studies showed that cytokine-directed agents such as IL-6 and IL-1 inhibitors may effective in the treatment of cytokine storm syndromes, including macrophage activation syndrome and cytokine release syndrome. Anti-human granulocyte macrophage-colony stimulating factor (GM-CSF) monoclonal antibody included mavrilimumab and lenzilumab, is designed to prevent and treat cytokine storm. | Discussion |
| Limitations | 25 | Discuss limitations at study and outcome level (e.g., risk of bias), and at review-level (e.g., incomplete retrieval of identified research, reporting bias).  First, a lack of RCTs limited our analyses. Some included studies were case reports or series, had no proper control groups. Meanwhile some articles that are not accessible to full texts and those in languages other than Chines and English were excluded from the analysis. This might have led to overlook some critical findings or observations.  In addition, in this study, the sofa scores or related indicators of some patients included in the study are median or mean, so not all patients are septic patients, but the results of this population also reflect a trend problem. Because some patients may be or will be in a state of sepsis.  Thirdly, we found that most patients use antiviral drugs, glucocorticoids, immunoglobulins, plasma, broad-spectrum antibiotics and other drugs at the same time. We can't rule out the impact of these drugs on the disease. | Discussion |
| Conclusions | 26 | Provide a general interpretation of the results in the context of other evidence, and implications for future research.  The results of systematic review showed that tocilizumab, sarilumab, anakinra may reduce mortality of people with COVID-19(at the edge of sepsis), and tocilizumab does not significantly affect SAEs and secondary infections. However, given the limited clinical researches and low-quality evidence, this conclusion needs more clinical evidence to verify. In addition, so far, there is still no unified opinion on the timing, dosage, usage and applicable population of these drugs all over the world, which also adds to the uncertainty of the conclusion of this study. | Discussion |
| **FUNDING** | | |  |
| Funding | 27 | Describe sources of funding for the systematic review and other support (e.g., supply of data); role of funders for the systematic review.  None | Acknowledgments |

*From:*  Moher D, Liberati A, Tetzlaff J, Altman DG, The PRISMA Group (2009). Preferred Reporting Items for Systematic Reviews and Meta-Analyses: The PRISMA Statement. PLoS Med 6(6): e1000097. doi:10.1371/journal.pmed1000097

For more information, visit: **www.prisma-statement.org**.

Page 2 of 2

**Appendix 1. Search strategy**

| **PubMed search strategy – last searched on Aug 22, 2021** | | **Results** |
| --- | --- | --- |
| #1 | Search: ((((((((((tocilizumab[Title/Abstract]) OR (RHPM-1[Title/Abstract])) OR (RG-1569[Title/Abstract])) OR (R-1569[Title/Abstract])) OR (MSB11456[Title/Abstract])) OR (MSB-11456[Title/Abstract])) OR (atlizumab[Title/Abstract])) OR (monoclonal antibody, MRA[Title/Abstract])) OR (RO-4877533[Title/Abstract])) OR (Actemra[Title/Abstract])) OR (Roactemra[Title/Abstract]) | 4536 |
| #2 | Search: ((((((((((((((((((((((((((((((((((COVID 19[Title/Abstract]) OR (COVID-19 Virus Disease[Title/Abstract])) OR (COVID 19 Virus Disease[Title/Abstract])) OR (COVID-19 Virus Diseases[Title/Abstract])) OR (Disease, COVID-19 Virus[Title/Abstract])) OR (Virus Disease, COVID-19[Title/Abstract])) OR (COVID-19 Virus Infection[Title/Abstract])) OR (COVID 19 Virus Infection[Title/Abstract])) OR (COVID-19 Virus Infections[Title/Abstract])) OR (Infection, COVID-19 Virus[Title/Abstract])) OR (Virus Infection, COVID-19[Title/Abstract])) OR (2019-nCoV Infection[Title/Abstract])) OR (2019 nCoV Infection[Title/Abstract])) OR (2019-nCoV Infections[Title/Abstract])) OR (Infection, 2019-nCoV[Title/Abstract])) OR (Coronavirus Disease-19[Title/Abstract])) OR (Coronavirus Disease 19[Title/Abstract])) OR (2019 Novel Coronavirus Disease[Title/Abstract])) OR (2019 Novel Coronavirus Infection[Title/Abstract])) OR (2019-nCoV Disease[Title/Abstract])) OR (2019 nCoV Disease[Title/Abstract])) OR (2019-nCoV Diseases[Title/Abstract])) OR (Disease, 2019-nCoV[Title/Abstract])) OR (COVID19[Title/Abstract])) OR (Coronavirus Disease 2019[Title/Abstract])) OR (Disease 2019, Coronavirus[Title/Abstract])) OR (SARS Coronavirus 2 Infection[Title/Abstract])) OR (SARS-CoV-2 Infection[Title/Abstract])) OR (Infection, SARS-CoV-2[Title/Abstract])) OR (SARS CoV 2 Infection[Title/Abstract])) OR (SARS-CoV-2 Infections[Title/Abstract])) OR (COVID-19 Pandemic[Title/Abstract])) OR (COVID 19 Pandemic[Title/Abstract])) OR (COVID-19 Pandemics[Title/Abstract])) OR (Pandemic, COVID-19[Title/Abstract]) | 151,606 |
| #3 | Search: (((((((SARS Coronavirus 2 VUI-202012-01[Title/Abstract]) OR (SARS-CoV-2 VUI-202012-01[Title/Abstract])) OR (Severe Acute Respiratory Syndrome Coronavirus 2 variant 202012 01[Title/Abstract])) OR (SARS-Cov-2 variant VUI-202012 01[Title/Abstract])) OR (COVID-19 Virus variant 202012-01[Title/Abstract])) OR (COVID-19 Virus variant 202012 01[Title/Abstract])) OR (Severe Acute Respiratory Syndrome Coronavirus 2 variant 202012-01[Title/Abstract])) OR (SARS Coronavirus 2 variant VUI-202012-01[Title/Abstract]) | 50 |
| #4 | Search: (((((((((((((((((((Coronavirus Disease 2019 Virus[Title/Abstract]) OR (2019 Novel Coronavirus[Title/Abstract])) OR (2019 Novel Coronaviruses[Title/Abstract])) OR (Coronavirus, 2019 Novel[Title/Abstract])) OR (Novel Coronavirus, 2019[Title/Abstract])) OR (Wuhan Seafood Market Pneumonia Virus[Title/Abstract])) OR (SARS-CoV-2 Virus[Title/Abstract])) OR (SARS CoV 2 Virus[Title/Abstract])) OR (SARS-CoV-2 Viruses[Title/Abstract])) OR (Virus, SARS-CoV-2[Title/Abstract])) OR (2019-nCoV[Title/Abstract])) OR (COVID-19 Virus[Title/Abstract])) OR (COVID 19 Virus[Title/Abstract])) OR (COVID-19 Viruses[Title/Abstract])) OR (Virus, COVID-19[Title/Abstract])) OR (Wuhan Coronavirus[Title/Abstract])) OR (Coronavirus, Wuhan[Title/Abstract])) OR (SARS Coronavirus 2[Title/Abstract])) OR (Coronavirus 2, SARS[Title/Abstract])) OR (Severe Acute Respiratory Syndrome Coronavirus 2[Title/Abstract]) | 23,440 |
| #5 | Search: ((((((((((((IL1 Febrile Inhibitor[Title/Abstract]) OR (Febrile Inhibitor, IL1[Title/Abstract])) OR (IL-1Ra[Title/Abstract])) OR (Urine-Derived IL1 Inhibitor[Title/Abstract])) OR (IL1 Inhibitor, Urine-Derived[Title/Abstract])) OR (Urine Derived IL1 Inhibitor[Title/Abstract])) OR (IL-1 Inhibitor, Urine[Title/Abstract])) OR (IL 1 Inhibitor, Urine[Title/Abstract])) OR (Urine IL-1 Inhibitor[Title/Abstract])) OR (interleukin 1 Inhibitor, Urine[Title/Abstract])) OR (antral[Title/Abstract])) OR (Kineret[Title/Abstract])) OR (Anakinra[Title/Abstract]) | 23,596 |
| #6 | Search: ((((((((CLLB8[Title/Abstract]) OR (cClB8 monoclonal antibody[Title/Abstract])) OR (sylvan[Title/Abstract])) OR (CNTO-328[Title/Abstract])) OR (monoclonal antibody CNTO-328[Title/Abstract])) OR (CNTO 328 monoclonal antibody[Title/Abstract])) OR (monoclonal antibody CNTO 328[Title/Abstract])) OR (monoclonal antibody CNTO328[Title/Abstract])) OR (siltuximab) | 353 |
| #7 | Search: (((((SAR-153191[Title/Abstract]) OR (SAR153191[Title/Abstract])) OR (Kevzara[Title/Abstract])) OR (REGN-88[Title/Abstract])) OR (REGN88[Title/Abstract])) OR (sarilumab[Title/Abstract]) | 195 |
| #8 | Search: (((Mavrilimumab) OR (CAM-3001[Title/Abstract])) OR (CAM3001[Title/Abstract])) OR (Antirheumatic Agents[Title/Abstract]) | 562 |
| #9 | Search: **(KB003) OR (lenzilumab)** | 17 |
| #11 | Search: (#2 OR #3 OR #4) AND (#9) | 10 |
| #12 | Search: (#2 OR #3 OR #4) AND (#8) | 80 |
| #13 | Search: (#2 OR #3 OR #4) AND (#7) | 68 |
| #14 | Search: (#2 OR #3 OR #4) AND (#6) | 36 |
| #15 | Search: (#2 OR #3 OR #4) AND (#5) | 222 |
| #16 | Search: (#2 OR #3 OR #4) AND (#1) | 1,103 |

| **MedRxiv & BioRxiv search strategy – last searched on Jun 10, 2021** | | **Results** |
| --- | --- | --- |
| #1 | abstract or title "tocilizumab" (match all words) and posted between "01 Jan, 2020 and 10 Jun, 2021" | 103 |
| #2 | abstract or title "anakinra" (match all words) and posted between "01 Jan, 2020 and 10 Jun, 2021" | 18 |
| #3 | abstract or title "sarilumab" (match all words) and posted between "01 Jan, 2020 and 10 Jun, 2021" | 8 |
| #4 | abstract or title "siltuximab" (match all words) and posted between "01 Jan, 2020 and 10 Jun, 2021" | 2 |
| #5 | abstract or title "Mavrilimumab" (match all words) | 0 |
| #6 | abstract or title "Lenzilumab" (match all words) | 1 |

| **Cochrane Library search strategy – last searched on Jun 10, 2021** | | **Results** |
| --- | --- | --- |
| #1 | (Tocilizumab):ti,ab,kw OR (atlizumab):ti,ab,kw OR (Actemra):ti,ab,kw OR (RoActemra):ti,ab,kw | 1170 |
| #2 | (Sarilumab):ti,ab,kw OR (Kevzara):ti,ab,kw OR (Siltuximab):ti,ab,kw OR (Sylvant):ti,ab,kw OR (CNTO-328):ti,ab,kw | 307 |
| #3 | (2019 nCoV):ti,ab,kw OR (COVID-19):ti,ab,kw OR (COVID-19 Virus Disease):ti,ab,kw OR (SARS-CoV-2):ti,ab,kw OR (coronavirus):ti,ab,kw | 3650 |
| #4 | (Mavrilimumab):ti,ab,kw OR (lenzilumab):ti,ab,kw | 54 |
| #5 | (Anakinra):ti,ab,kw OR (Kineret):ti,ab,kw OR (Antril):ti,ab,kw OR (IL-1 Inhibitor):ti,ab,kw | 567 |
| #6 | #3 AND #1 | 110 |
| #7 | #3 AND #2 | 33 |
| #8 | #3 AND #4 | 14 |
| #9 | #3 AND #5 | 39 |

| **Embase search strategy – last searched on Jun 10, 2021** | | **Results** |
| --- | --- | --- |
| #1 | 'tocilizumab'/exp OR atlizumab:ti,ab,kw OR actemra:ti,ab,kw OR roactemra:ti,ab,kw | 14659 |
| #2 | 'coronavirus disease 2019'/exp OR 'coronavirus disease 2019'/exp OR 'severe acute respiratory syndrome coronavirus 2'/exp | 77847 |
| #3 | ‘sarilumab‘/exp OR kevzara:ti,ab,kw | 848 |
| #4 | ‘siltuximab’/exp OR sylvant:ti,ab,kw OR 'cnto 328':ti,ab,kw) | 862 |
| #5 | ‘mavrilimumab’/exp | 127 |
| #6 | ‘lenzilumab’/exp | 55 |
| #7 | ‘anakinra’/exp OR ‘kineret’:ab,ti,kw OR ‘antril’:ab,ti,kw OR ‘il-1 inhibitor’:ab,ti,kw | 9144 |
| #8 | #2 AND #1 | 1915 |
| #9 | #2 AND #3 | 301 |
| #10 | #2 AND #4 | 112 |
| #11 | #2 AND #5 | 21 |
| #12 | #2 AND #6 | 20 |
| #13 | #2 AND #7 | 391 |

CNKI: 0

Wangfang Database: 0
