# Supplementary material for: Specific Interleukin-1 Inhibitors, Specific Interleukin-6 Inhibitors, and GM-CSF Blockades for COVID-19 (at the Edge of Sepsis): A Systematic Review
Source: Front Pharmacol. 2022 Jan 21;12:804250. doi: 10.3389/fphar.2021.804250 (PMC8815770; doi:10.3389/fphar.2021.804250)
Supplement: Supplementary file 2 [file DataSheet2.docx]

**Supplementary appendix**

| **Supplementary figure S1. Risk of bias of RCTs** | **1** |
| --- | --- |
| **Supplementary table 1. NOS assessment results of cohort studies for mortality** | **2-17** |
| **Supplementary table 2. NOS assessment results of cohort studies for safety outcome (SAEs)** | **18-25** |
| **Supplementary table 3. NOS assessment results of cohort studies for safety(secondary infections)** | **25-35** |
| **Supplementary table 4. NOS assessment results of case-control studies for mortality** | **36-37** |
| **Supplementary table 4. NOS assessment results of case-control studies for safety(SAEs)** | **38-39** |
| **Supplementary table** **6. NOS assessment results of case-control studies for safety(secondary infections)** | **40** |
| **Supplementary table 7. Quality Assessment for Case Series** | **41-42** |
| **Supplementary table 8. Grade of evidence** | **43-45** |

**figure S1 Risk of bias of RCTs**

**
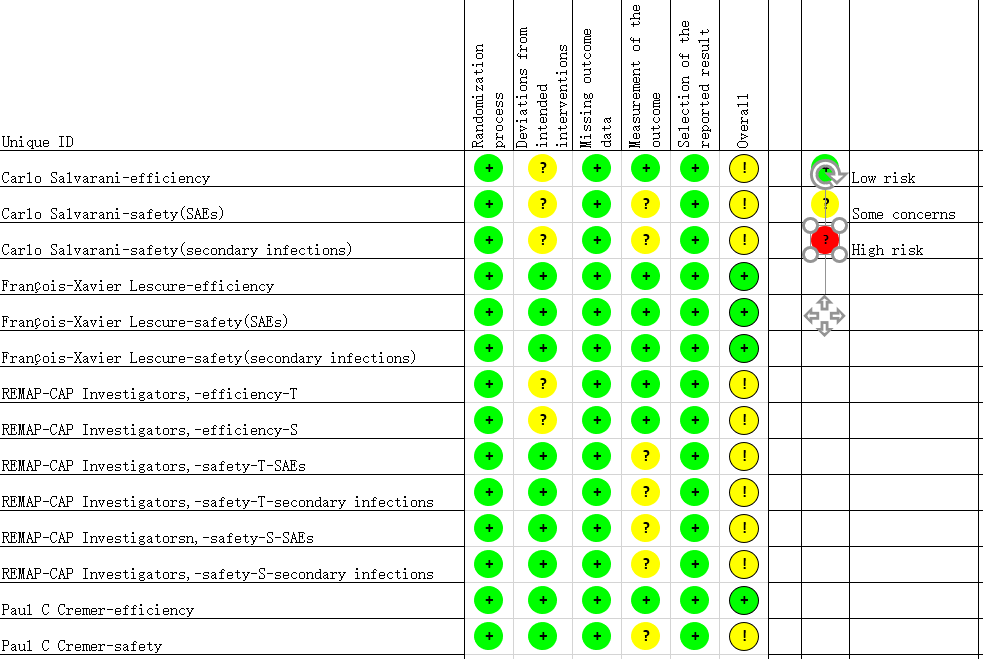
**

**Supplementary table** **1. NOS assessment results of cohort studies for mortality**

| **Study name:** **Lorenzo M. Canziani**** | |
| --- | --- |
| **Items** | **Response options** |
| Selection | |
| *Representativeness of the exposed cohort？* | *** |
| *Selection of the non exposed cohort* | *** |
| *Ascertainment of exposure* | *** |
| *Demonstration that outcome of interest was not present at start of study* | *** |
| Comparability | |
| *Comparability of cohorts on the basis of the design or analysis* | *** |
| Outcome | |
| *Assessment of outcome* | *** |
| *Was follow-up long enough for outcomes to occur* | *** |
| *Adequacy of follow up of cohorts* |  |

Cohort studies with scores of 0-3, 4-6, 7-9 were, respectively, considered as low, moderate, and high quality.

High quality

| **Study name:** **Matthew J. Fisher**** | |
| --- | --- |
| **Items** | **Response options** |
| Selection | |
| *Representativeness of the exposed cohort？* | *** |
| *Selection of the non exposed cohort* | *** |
| *Ascertainment of exposure* | *** |
| *Demonstration that outcome of interest was not present at start of study* | *** |
| Comparability | |
| *Comparability of cohorts on the basis of the design or analysis* |  |
| Outcome | |
| *Assessment of outcome* | *** |
| *Was follow-up long enough for outcomes to occur* | *** |
| *Adequacy of follow up of cohorts* |  |

Moderate quality

| **Study name:** **Corrado Campochiaroa**** | |
| --- | --- |
| **Items** | **Response options** |
| Selection | |
| *Representativeness of the exposed cohort？* | *** |
| *Selection of the non exposed cohort* | *** |
| *Ascertainment of exposure* | *** |
| *Demonstration that outcome of interest was not present at start of study* | *** |
| Comparability | |
| *Comparability of cohorts on the basis of the design or analysis* |  |
| Outcome | |
| *Assessment of outcome* | *** |
| *Was follow-up long enough for outcomes to occur* | *** |
| *Adequacy of follow up of cohorts* |  |

Moderate quality

| **Study name:** **M. Cristina Vazquez Guillamet**** | |
| --- | --- |
| **Items** | **Response options** |
| Selection | |
| *Representativeness of the exposed cohort？* |  |
| *Selection of the non exposed cohort* |  |
| *Ascertainment of exposure* | *** |
| *Demonstration that outcome of interest was not present at start of study* | *** |
| Comparability | |
| *Comparability of cohorts on the basis of the design or analysis* |  |
| Outcome | |
| *Assessment of outcome* | *** |
| *Was follow-up long enough for outcomes to occur* | *** |
| *Adequacy of follow up of cohorts* |  |

Moderate quality

| **Study name:** **Prabalini Rajendram**** | |
| --- | --- |
| **Items** | **Response options** |
| Selection | |
| *Representativeness of the exposed cohort？* | *** |
| *Selection of the non exposed cohort* | *** |
| *Ascertainment of exposure* | *** |
| *Demonstration that outcome of interest was not present at start of study* | *** |
| Comparability | |
| *Comparability of cohorts on the basis of the design or analysis* | *** |
| Outcome | |
| *Assessment of outcome* | *** |
| *Was follow-up long enough for outcomes to occur* | *** |
| *Adequacy of follow up of cohorts* | *** |

High quality

| **Study name:** **Edmund Huang**** | |
| --- | --- |
| **Items** | **Response options** |
| Selection | |
| *Representativeness of the exposed cohort？* | *** |
| *Selection of the non exposed cohort* | *** |
| *Ascertainment of exposure* | *** |
| *Demonstration that outcome of interest was not present at start of study* | *** |
| Comparability | |
| *Comparability of cohorts on the basis of the design or analysis* |  |
| Outcome | |
| *Assessment of outcome* | *** |
| *Was follow-up long enough for outcomes to occur* | *** |
| *Adequacy of follow up of cohorts* | *** |

High quality

| **Study name:** **Edmund Huang**** | |
| --- | --- |
| **Items** | **Response options** |
| Selection | |
| *Representativeness of the exposed cohort？* | *** |
| *Selection of the non exposed cohort* | *** |
| *Ascertainment of exposure* | *** |
| *Demonstration that outcome of interest was not present at start of study* | *** |
| Comparability | |
| *Comparability of cohorts on the basis of the design or analysis* |  |
| Outcome | |
| *Assessment of outcome* | *** |
| *Was follow-up long enough for outcomes to occur* | *** |
| *Adequacy of follow up of cohorts* | *** |

High quality

| **Study name:** **Zaid Saffo**** | |
| --- | --- |
| **Items** | **Response options** |
| Selection | |
| *Representativeness of the exposed cohort？* | *** |
| *Selection of the non exposed cohort* | *** |
| *Ascertainment of exposure* | *** |
| *Demonstration that outcome of interest was not present at start of study* | *** |
| Comparability | |
| *Comparability of cohorts on the basis of the design or analysis* |  |
| Outcome | |
| *Assessment of outcome* | *** |
| *Was follow-up long enough for outcomes to occur* | *** |
| *Adequacy of follow up of cohorts* |  |

Moderate quality

| **Study name:** Emily C Somers****** | |
| --- | --- |
| **Items** | **Response options** |
| Selection | |
| *Representativeness of the exposed cohort？* | *** |
| *Selection of the non exposed cohort* | *** |
| *Ascertainment of exposure* | *** |
| *Demonstration that outcome of interest was not present at start of study* | *** |
| Comparability | |
| *Comparability of cohorts on the basis of the design or analysis* | *** |
| Outcome | |
| *Assessment of outcome* | *** |
| *Was follow-up long enough for outcomes to occur* | *** |
| *Adequacy of follow up of cohorts* | *** |

High quality

| **Study name:** Shari B. Brosnahan****** | |
| --- | --- |
| **Items** | **Response options** |
| Selection | |
| *Representativeness of the exposed cohort？* | *** |
| *Selection of the non exposed cohort* |  |
| *Ascertainment of exposure* | *** |
| *Demonstration that outcome of interest was not present at start of study* | *** |
| Comparability | |
| *Comparability of cohorts on the basis of the design or analysis* |  |
| Outcome | |
| *Assessment of outcome* | *** |
| *Was follow-up long enough for outcomes to occur* | *** |
| *Adequacy of follow up of cohorts* |  |

Moderate quality

| **Study name:** Giacomo De Luca****** | |
| --- | --- |
| **Items** | **Response options** |
| Selection | |
| *Representativeness of the exposed cohort？* | *** |
| *Selection of the non exposed cohort* | *** |
| *Ascertainment of exposure* | *** |
| *Demonstration that outcome of interest was not present at start of study* | *** |
| Comparability | |
| *Comparability of cohorts on the basis of the design or analysis* |  |
| Outcome | |
| *Assessment of outcome* | *** |
| *Was follow-up long enough for outcomes to occur* | *** |
| *Adequacy of follow up of cohorts* |  |

Moderate quality

| **Study name:** Emanuel Della- Torre****** | |
| --- | --- |
| **Items** | **Response options** |
| Selection | |
| *Representativeness of the exposed cohort？* | *** |
| *Selection of the non exposed cohort* | *** |
| *Ascertainment of exposure* | *** |
| *Demonstration that outcome of interest was not present at start of study* | *** |
| Comparability | |
| *Comparability of cohorts on the basis of the design or analysis* |  |
| Outcome | |
| *Assessment of outcome* | *** |
| *Was follow-up long enough for outcomes to occur* | *** |
| *Adequacy of follow up of cohorts* |  |

Moderate quality

| **Study name:** Giorgio Bozzi****** | |
| --- | --- |
| **Items** | **Response options** |
| Selection | |
| *Representativeness of the exposed cohort？* | *** |
| *Selection of the non exposed cohort* | *** |
| *Ascertainment of exposure* | *** |
| *Demonstration that outcome of interest was not present at start of study* | *** |
| Comparability | |
| *Comparability of cohorts on the basis of the design or analysis* |  |
| Outcome | |
| *Assessment of outcome* | *** |
| *Was follow-up long enough for outcomes to occur* | *** |
| *Adequacy of follow up of cohorts* |  |

Moderate quality

| **Study name:** **Marco Franzetti**** | |
| --- | --- |
| **Items** | **Response options** |
| Selection | |
| *Representativeness of the exposed cohort？* | *** |
| *Selection of the non exposed cohort* | *** |
| *Ascertainment of exposure* | *** |
| *Demonstration that outcome of interest was not present at start of study* | *** |
| Comparability | |
| *Comparability of cohorts on the basis of the design or analysis* | *** |
| Outcome | |
| *Assessment of outcome* | *** |
| *Was follow-up long enough for outcomes to occur* | *** |
| *Adequacy of follow up of cohorts* |  |

High quality

| **Study name:** **Evdoxia Kyriazopoulou**** | |
| --- | --- |
| **Items** | **Response options** |
| Selection | |
| *Representativeness of the exposed cohort？* | *** |
| *Selection of the non exposed cohort* | *** |
| *Ascertainment of exposure* | *** |
| *Demonstration that outcome of interest was not present at start of study* | *** |
| Comparability | |
| *Comparability of cohorts on the basis of the design or analysis* | *** |
| Outcome | |
| *Assessment of outcome* | *** |
| *Was follow-up long enough for outcomes to occur* | *** |
| *Adequacy of follow up of cohorts* | *** |

High quality

| **Study name:** **Giulio Cavalli**** | |
| --- | --- |
| **Items** | **Response options** |
| Selection | |
| *Representativeness of the exposed cohort？* | *** |
| *Selection of the non exposed cohort* |  |
| *Ascertainment of exposure* | *** |
| *Demonstration that outcome of interest was not present at start of study* | *** |
| Comparability | |
| *Comparability of cohorts on the basis of the design or analysis* |  |
| Outcome | |
| *Assessment of outcome* | *** |
| *Was follow-up long enough for outcomes to occur* | *** |
| *Adequacy of follow up of cohorts* |  |

Moderate quality

**Supplementary table** **2. NOS assessment results of cohort studies for safety outcome(SAEs)**

| **Study name:** **Lorenzo M. Canziani**** | |
| --- | --- |
| **Items** | **Response options** |
| Selection | |
| *Representativeness of the exposed cohort？* | *** |
| *Selection of the non exposed cohort* | *** |
| *Ascertainment of exposure* | *** |
| *Demonstration that outcome of interest was not present at start of study* |  |
| Comparability | |
| *Comparability of cohorts on the basis of the design or analysis* | *** |
| Outcome | |
| *Assessment of outcome* | *** |
| *Was follow-up long enough for outcomes to occur* |  |
| *Adequacy of follow up of cohorts* |  |

Cohort studies with scores of 0-3, 4-6, 7-9 were, respectively, considered as low, moderate, and high quality.

Moderate quality

| **Study name:** **Corrado Campochiaroa **** | |
| --- | --- |
| **Items** | **Response options** |
| Selection | |
| *Representativeness of the exposed cohort？* | *** |
| *Selection of the non exposed cohort* | *** |
| *Ascertainment of exposure* | *** |
| *Demonstration that outcome of interest was not present at start of study* | *** |
| Comparability | |
| *Comparability of cohorts on the basis of the design or analysis* |  |
| Outcome | |
| *Assessment of outcome* |  |
| *Was follow-up long enough for outcomes to occur* |  |
| *Adequacy of follow up of cohorts* |  |

Moderate quality

| **Study name:** **M. Cristina Vazquez Guillamet**** | |
| --- | --- |
| **Items** | **Response options** |
| Selection | |
| *Representativeness of the exposed cohort？* |  |
| *Selection of the non exposed cohort* |  |
| *Ascertainment of exposure* | *** |
| *Demonstration that outcome of interest was not present at start of study* |  |
| Comparability | |
| *Comparability of cohorts on the basis of the design or analysis* |  |
| Outcome | |
| *Assessment of outcome* | *** |
| *Was follow-up long enough for outcomes to occur* |  |
| *Adequacy of follow up of cohorts* |  |

Low quality

| **Study name:** **Prabalini Rajendram**** | |
| --- | --- |
| **Items** | **Response options** |
| Selection | |
| *Representativeness of the exposed cohort？* | *** |
| *Selection of the non exposed cohort* | *** |
| *Ascertainment of exposure* | *** |
| *Demonstration that outcome of interest was not present at start of study* |  |
| Comparability | |
| *Comparability of cohorts on the basis of the design or analysis* | *** |
| Outcome | |
| *Assessment of outcome* | *** |
| *Was follow-up long enough for outcomes to occur* |  |
| *Adequacy of follow up of cohorts* |  |

Moderate quality

| **Study name:** **Zaid Saffo**** | |
| --- | --- |
| **Items** | **Response options** |
| Selection | |
| *Representativeness of the exposed cohort？* | *** |
| *Selection of the non exposed cohort* | *** |
| *Ascertainment of exposure* | *** |
| *Demonstration that outcome of interest was not present at start of study* |  |
| Comparability | |
| *Comparability of cohorts on the basis of the design or analysis* |  |
| Outcome | |
| *Assessment of outcome* | *** |
| *Was follow-up long enough for outcomes to occur* |  |
| *Adequacy of follow up of cohorts* | *** |

Moderate quality

| **Study name:** Emily C Somers****** | |
| --- | --- |
| **Items** | **Response options** |
| Selection | |
| *Representativeness of the exposed cohort？* | *** |
| *Selection of the non exposed cohort* | *** |
| *Ascertainment of exposure* | *** |
| *Demonstration that outcome of interest was not present at start of study* | *** |
| Comparability | |
| *Comparability of cohorts on the basis of the design or analysis* | *** |
| Outcome | |
| *Assessment of outcome* | *** |
| *Was follow-up long enough for outcomes to occur* | *** |
| *Adequacy of follow up of cohorts* | *** |

High quality

| **Study name:** Shari B. Brosnahan****** | |
| --- | --- |
| **Items** | **Response options** |
| Selection | |
| *Representativeness of the exposed cohort？* | *** |
| *Selection of the non exposed cohort* |  |
| *Ascertainment of exposure* | *** |
| *Demonstration that outcome of interest was not present at start of study* |  |
| Comparability | |
| *Comparability of cohorts on the basis of the design or analysis* |  |
| Outcome | |
| *Assessment of outcome* | *** |
| *Was follow-up long enough for outcomes to occur* |  |
| *Adequacy of follow up of cohorts* |  |

Low quality

**Supplementary table** **3. NOS assessment results of cohort studies for safety(secondary infections)**

| **Study name:** **Lorenzo M. Canziani**** | |
| --- | --- |
| **Items** | **Response options** |
| Selection | |
| *Representativeness of the exposed cohort？* | *** |
| *Selection of the non exposed cohort* | *** |
| *Ascertainment of exposure* | *** |
| *Demonstration that outcome of interest was not present at start of study* |  |
| Comparability | |
| *Comparability of cohorts on the basis of the design or analysis* | *** |
| Outcome | |
| *Assessment of outcome* | *** |
| *Was follow-up long enough for outcomes to occur* |  |
| *Adequacy of follow up of cohorts* |  |

Cohort studies with scores of 0-3, 4-6, 7-9 were, respectively, considered as low, moderate, and high quality.

Moderate quality

| **Study name:** **Matthew J. Fisher**** | |
| --- | --- |
| **Items** | **Response options** |
| Selection | |
| *Representativeness of the exposed cohort？* | *** |
| *Selection of the non exposed cohort* | *** |
| *Ascertainment of exposure* | *** |
| *Demonstration that outcome of interest was not present at start of study* |  |
| Comparability | |
| *Comparability of cohorts on the basis of the design or analysis* |  |
| Outcome | |
| *Assessment of outcome* |  |
| *Was follow-up long enough for outcomes to occur* |  |
| *Adequacy of follow up of cohorts* |  |

Low quality

| **Study name:** **Corrado Campochiaroa **** | |
| --- | --- |
| **Items** | **Response options** |
| Selection | |
| *Representativeness of the exposed cohort？* | *** |
| *Selection of the non exposed cohort* | *** |
| *Ascertainment of exposure* | *** |
| *Demonstration that outcome of interest was not present at start of study* | *** |
| Comparability | |
| *Comparability of cohorts on the basis of the design or analysis* |  |
| Outcome | |
| *Assessment of outcome* |  |
| *Was follow-up long enough for outcomes to occur* |  |
| *Adequacy of follow up of cohorts* |  |

Moderate quality

| **Study name:** **M. Cristina Vazquez Guillamet**** | |
| --- | --- |
| **Items** | **Response options** |
| Selection | |
| *Representativeness of the exposed cohort？* |  |
| *Selection of the non exposed cohort* |  |
| *Ascertainment of exposure* | *** |
| *Demonstration that outcome of interest was not present at start of study* |  |
| Comparability | |
| *Comparability of cohorts on the basis of the design or analysis* |  |
| Outcome | |
| *Assessment of outcome* | *** |
| *Was follow-up long enough for outcomes to occur* |  |
| *Adequacy of follow up of cohorts* |  |

Low quality

| **Study name:** **Prabalini Rajendram**** | |
| --- | --- |
| **Items** | **Response options** |
| Selection | |
| *Representativeness of the exposed cohort？* | *** |
| *Selection of the non exposed cohort* | *** |
| *Ascertainment of exposure* | *** |
| *Demonstration that outcome of interest was not present at start of study* |  |
| Comparability | |
| *Comparability of cohorts on the basis of the design or analysis* | *** |
| Outcome | |
| *Assessment of outcome* | *** |
| *Was follow-up long enough for outcomes to occur* |  |
| *Adequacy of follow up of cohorts* |  |

Moderate quality

| **Study name:** **Edmund Huang**** | |
| --- | --- |
| **Items** | **Response options** |
| Selection | |
| *Representativeness of the exposed cohort？* | *** |
| *Selection of the non exposed cohort* | *** |
| *Ascertainment of exposure* | *** |
| *Demonstration that outcome of interest was not present at start of study* |  |
| Comparability | |
| *Comparability of cohorts on the basis of the design or analysis* |  |
| Outcome | |
| *Assessment of outcome* | *** |
| *Was follow-up long enough for outcomes to occur* |  |
| *Adequacy of follow up of cohorts* | *** |

moderate quality

| **Study name:** **Zaid Saffo**** | |
| --- | --- |
| **Items** | **Response options** |
| Selection | |
| *Representativeness of the exposed cohort？* | *** |
| *Selection of the non exposed cohort* | *** |
| *Ascertainment of exposure* | *** |
| *Demonstration that outcome of interest was not present at start of study* |  |
| Comparability | |
| *Comparability of cohorts on the basis of the design or analysis* |  |
| Outcome | |
| *Assessment of outcome* | *** |
| *Was follow-up long enough for outcomes to occur* |  |
| *Adequacy of follow up of cohorts* | *** |

Moderate quality

| **Study name:** Emily C Somers****** | |
| --- | --- |
| **Items** | **Response options** |
| Selection | |
| *Representativeness of the exposed cohort？* | *** |
| *Selection of the non exposed cohort* | *** |
| *Ascertainment of exposure* | *** |
| *Demonstration that outcome of interest was not present at start of study* | *** |
| Comparability | |
| *Comparability of cohorts on the basis of the design or analysis* | *** |
| Outcome | |
| *Assessment of outcome* | *** |
| *Was follow-up long enough for outcomes to occur* | *** |
| *Adequacy of follow up of cohorts* | *** |

High quality

| **Study name:** Shari B. Brosnahan****** | |
| --- | --- |
| **Items** | **Response options** |
| Selection | |
| *Representativeness of the exposed cohort？* | *** |
| *Selection of the non exposed cohort* |  |
| *Ascertainment of exposure* | *** |
| *Demonstration that outcome of interest was not present at start of study* |  |
| Comparability | |
| *Comparability of cohorts on the basis of the design or analysis* |  |
| Outcome | |
| *Assessment of outcome* | *** |
| *Was follow-up long enough for outcomes to occur* |  |
| *Adequacy of follow up of cohorts* |  |

Low quality

| **Study name:** Giacomo De Luca****** | |
| --- | --- |
| **Items** | **Response options** |
| Selection | |
| *Representativeness of the exposed cohort？* | *** |
| *Selection of the non exposed cohort* | *** |
| *Ascertainment of exposure* | *** |
| *Demonstration that outcome of interest was not present at start of study* |  |
| Comparability | |
| *Comparability of cohorts on the basis of the design or analysis* |  |
| Outcome | |
| *Assessment of outcome* | *** |
| *Was follow-up long enough for outcomes to occur* | *** |
| *Adequacy of follow up of cohorts* |  |

Moderate quality

| **Study name:** Emanuel Della- Torre****** | |
| --- | --- |
| **Items** | **Response options** |
| Selection | |
| *Representativeness of the exposed cohort？* | *** |
| *Selection of the non exposed cohort* | *** |
| *Ascertainment of exposure* | *** |
| *Demonstration that outcome of interest was not present at start of study* |  |
| Comparability | |
| *Comparability of cohorts on the basis of the design or analysis* |  |
| Outcome | |
| *Assessment of outcome* | *** |
| *Was follow-up long enough for outcomes to occur* |  |
| *Adequacy of follow up of cohorts* |  |

Moderate quality

**Supplementary table** 4**. NOS assessment results of case-control studies for mortality**

| **Study name:** Galván-Román, J. M****** | |
| --- | --- |
| **Items** | **Response options** |
| Selection | |
| *Is the case definition adequate?？* | *** |
| *Representativeness of the cases* | *** |
| *Selection of controls* |  |
| *Definition of controls* | *** |
| Comparability | |
| *Comparability of cohorts on the basis of the design or analysis* |  |
| Exposure | |
| *Ascertainment of exposure* | *** |
| *Same method of ascertainment for cases and controls* |  |
| *Non-Response rate* |  |

Moderate quality

| **Study name:** Francesco Menzella****** | |
| --- | --- |
| **Items** | **Response options** |
| Selection | |
| *Is the case definition adequate?？* | *** |
| *Representativeness of the cases* | *** |
| *Selection of controls* |  |
| *Definition of controls* | *** |
| Comparability | |
| *Comparability of cohorts on the basis of the design or analysis* |  |
| Exposure | |
| *Ascertainment of exposure* | *** |
| *Same method of ascertainment for cases and controls* |  |
| *Non-Response rate* |  |

Moderate quality

**Supplementary table** **5. NOS assessment results of case-control studies for safety(SAEs)**

| **Study name:** Galván-Román, J. M****** | |
| --- | --- |
| **Items** | **Response options** |
| Selection | |
| *Is the case definition adequate?？* | *** |
| *Representativeness of the cases* | *** |
| *Selection of controls* |  |
| *Definition of controls* | *** |
| Comparability | |
| *Comparability of cohorts on the basis of the design or analysis* |  |
| Exposure | |
| *Ascertainment of exposure* | *** |
| *Same method of ascertainment for cases and controls* |  |
| *Non-Response rate* |  |

Moderate quality

| **Study name:** Francesco Menzella****** | |
| --- | --- |
| **Items** | **Response options** |
| Selection | |
| *Is the case definition adequate?？* | *** |
| *Representativeness of the cases* | *** |
| *Selection of controls* |  |
| *Definition of controls* |  |
| Comparability | |
| *Comparability of cohorts on the basis of the design or analysis* |  |
| Exposure | |
| *Ascertainment of exposure* | *** |
| *Same method of ascertainment for cases and controls* |  |
| *Non-Response rate* |  |

Low quality

**Supplementary table** **6. NOS assessment results of case-control studies for safety(secondary infections)**

| **Study name:** Francesco Menzella****** | |
| --- | --- |
| **Items** | **Response options** |
| Selection | |
| *Is the case definition adequate?？* | *** |
| *Representativeness of the cases* | *** |
| *Selection of controls* |  |
| *Definition of controls* |  |
| Comparability | |
| *Comparability of cohorts on the basis of the design or analysis* |  |
| Exposure | |
| *Ascertainment of exposure* | *** |
| *Same method of ascertainment for cases and controls* |  |
| *Non-Response rate* |  |

Low quality

**Supplementary Table 7 Quality Assessment for Case Series**

| Author | Case series collected in more than one center | Is the hypothesis/aim/objective of the study clearly described? | Are the inclusion and exclusion criteria (case definition) clearly reported? | Is there a clear definition of the outcomes reported? | Were data collected prospectively? | Is there an explicit statement that patients were recruited consecutively? | Are the main findings of the study clearly described? | Are outcomes stratified? | scores |
| --- | --- | --- | --- | --- | --- | --- | --- | --- | --- |
| Matt G. McKenzie | 1 | 1 | 1 | 1 | 0 | 0 | 1 | 1 | 6 |
| Fahmi A. AL-Kaf | 0 | 0 | 1 | 1 | 0 | 0 | 1 | 0 | 3 |
| Ahmet Eroglu | 0 | 1 | 1 | 1 | 0 | 0 | 1 | 1 | 5 |
| Hiroshi Kataoka | 0 | 1 | 1 | 1 | 0 | 0 | 1 | 1 | 5 |
| Tomoo Kishaba | 0 | 1 | 1 | 1 | 0 | 0 | 1 | 1 | 5 |
| Nophol Leelayuwatanakul | 0 | 1 | 1 | 1 | 0 | 0 | 1 | 1 | 5 |
| Nicole Nourié | 0 | 1 | 1 | 1 | 0 | 0 | 1 | 1 | 5 |
| M. Ladna | 0 | 1 | 1 | 1 | 0 | 0 | 0 | 1 | 4 |
| Alexandra Cristina Senegaglia | 0 | 1 | 1 | 1 | 0 | 0 | 1 | 1 | 5 |
| Theerachai Thammathiwat | 0 | 1 | 1 | 1 | 0 | 0 | 1 | 1 | 5 |
| H`ector Corominas | 0 | 1 | 1 | 1 | 0 | 0 | 1 | 1 | 5 |
| Tatsuki Abe | 0 | 1 | 1 | 1 | 0 | 0 | 1 | 1 | 5 |
| Pratik A. Patel | 0 | 1 | 1 | 1 | 0 | 0 | 1 | 1 | 5 |
| Luca Bernardo | 0 | 1 | 1 | 1 | 0 | 0 | 1 | 0 | 4 |
| Jose A. Morillas | 0 | 1 | 1 | 1 | 0 | 0 | 1 | 0 | 4 |
| Marco Cascella | 0 | 1 | 1 | 1 | 0 | 0 | 1 | 1 | 5 |
| Giovanni Filocamo | 0 | 1 | 1 | 1 | 0 | 0 | 1 | 1 | 5 |
| Marco Franzetti | 0 | 1 | 1 | 1 | 0 | 0 | 1 | 1 | 5 |
| Ahmed Mady | 0 | 1 | 1 | 1 | 0 | 0 | 1 | 1 | 5 |

Yes=1, No =0. The total score:8. High quality:4~8. Low quality: < 4

**Supplementary table 8 Grade of evidence**

| **tocilizumab for COVID-19(at the rage of sepsis)** | | | | | | |
| --- | --- | --- | --- | --- | --- | --- |
| **Patient or population:** patients with [COVID-19(at the rage of sepsis)] **Settings:**  **Intervention:** tocilizumab/sarilumab/anakinra | | | | | | |
| **Outcomes** | **Illustrative comparative risks* (95% CI)** | | **Relative effect (95% CI)** | **No of Participants (studies)** | **Quality of the evidence (GRADE)** | **Comments** |
|  | Assumed risk | Corresponding risk |  |  |  |  |
|  | **Control** | **Tocilizumab** |  |  |  |  |
| **tocilizumab-RCT-mortality** | **Study population** | | **OR 0.71**  (0.52 to 0.97) | 870 (2 studies) | ⊕⊕⊝⊝ **low**^1,2^ |  |
|  | **311 per 1000** | **243 per 1000** (190 to 304) |  |  |  |  |
|  | **Moderate** | |  |  |  |  |
|  | **187 per 1000** | **140 per 1000** (107 to 182) |  |  |  |  |
| **tocilizumab-Non-RCT-mortality** | **Study population** | | **RR 0.68**  (0.55 to 0.84) | 1657 (11 studies) | ⊕⊝⊝⊝ **very low**^3,4^ |  |
|  | **445 per 1000** | **303 per 1000** (245 to 374) |  |  |  |  |
|  | **Moderate** | |  |  |  |  |
|  | **375 per 1000** | **255 per 1000** (206 to 315) |  |  |  |  |
| **tocilizumab-RCT-safety(SAEs)** | **Study population** | | **OR 0.87**  (0.38 to 2) | 878 (2 studies) | ⊕⊕⊝⊝ **low**^1,2^ |  |
|  | **28 per 1000** | **24 per 1000** (11 to 54) |  |  |  |  |
|  | **Moderate** | |  |  |  |  |
|  | **30 per 1000** | **26 per 1000** (12 to 58) |  |  |  |  |
| **tocilizumab-Non-RCT-Safety(SAEs)** | **Study population** | | **RR 1.18**  (0.83 to 1.68) | 469 (4 studies) | ⊕⊝⊝⊝ **very low**^4,5,6^ |  |
|  | **177 per 1000** | **209 per 1000** (147 to 297) |  |  |  |  |
|  | **Moderate** | |  |  |  |  |
|  | **209 per 1000** | **247 per 1000** (173 to 351) |  |  |  |  |
| **tocilizumab-Non-RCT-secondary infection** | **Study population** | | **RR 1.15**  (0.89 to 1.49) | 1511 (10 studies) | ⊕⊝⊝⊝ **very low**^4,5^ |  |
|  | **272 per 1000** | **312 per 1000** (242 to 405) |  |  |  |  |
|  | **Moderate** | |  |  |  |  |
|  | **257 per 1000** | **296 per 1000** (229 to 383) |  |  |  |  |
| **tocilizumab-RCT-safety(secondary infection)** | **Study population** | | **OR 0.71**  (0.06 to 8.75) | 878 (2 studies) | ⊕⊕⊝⊝ **low**^1,2^ |  |
|  | **9 per 1000** | **6 per 1000** (1 to 71) |  |  |  |  |
|  | **Moderate** | |  |  |  |  |
|  | **32 per 1000** | **23 per 1000** (2 to 224) |  |  |  |  |
| **sarilumab-RCT-mortality** | **Study population** | | **OR 0.65**  (0.36 to 1.2) | 699 (2 studies) | ⊕⊕⊝⊝ **low**^7,8^ |  |
|  | **310 per 1000** | **226 per 1000** (139 to 350) |  |  |  |  |
|  | **Moderate** | |  |  |  |  |
|  | **221 per 1000** | **156 per 1000** (93 to 254) |  |  |  |  |
| **anakinra-Non-RCT-mortality** | **Study population** | | **RR 0.47**  (0.34 to 0.66) | 492 (3 studies) | ⊕⊝⊝⊝ **very low**^9,10,11^ |  |
|  | **320 per 1000** | **150 per 1000** (109 to 211) |  |  |  |  |
|  | **Moderate** | |  |  |  |  |
|  | **346 per 1000** | **163 per 1000** (118 to 228) |  |  |  |  |
| *The basis for the **assumed risk** (e.g. the median control group risk across studies) is provided in footnotes. The **corresponding risk** (and its 95% confidence interval) is based on the assumed risk in the comparison group and the **relative effect** of the intervention (and its 95% CI).  **CI:** Confidence interval; **RR:** Risk ratio; **OR:** Odds ratio; | | | | | | |
| GRADE Working Group grades of evidence **High quality:** Further research is very unlikely to change our confidence in the estimate of effect.  **Moderate quality:** Further research is likely to have an important impact on our confidence in the estimate of effect and may change the estimate. **Low quality:** Further research is very likely to have an important impact on our confidence in the estimate of effect and is likely to change the estimate. **Very low quality:** We are very uncertain about the estimate. | | | | | | |
| ^1^ The risk of bias of RCTs were some concern. ^2^ There are too few randomized controlled studies to evaluate the information size and results, so we downgraded one point for imprecision. ^3^ Methodological quality of those studies were moderate, so we downgraded one point for risk of bias ^4^ Controlled non-randomised studies ^5^ Methodological quality of those studies were low, so we downgrade two point for risk of bias. ^6^ There are too few studies to evaluate the information size and results, so we downgraded one point for imprecision. ^7^ Risk of bias within these studies were some concerns, so we downgraded one point for risk of bias ^8^ There were too few studies to evaluate the information size and results, so we downgraded one point for imprecision ^9^ Methodological quality of those studies were moderate, so we downgraded one point for risk of bias. ^10^ There were too few studies to evaluate the information size and results, so we downgraded one point for imprecision. ^11^ RR 0.47 | | | | | | |
